# Comparative genomics of enterohemorrhagic *Escherichia coli* O145:H28 demonstrates a common evolutionary lineage with *Escherichia coli* O157:H7

**DOI:** 10.1186/1471-2164-15-17

**Published:** 2014-01-10

**Authors:** Kerry K Cooper, Robert E Mandrell, Jacqueline W Louie, Jonas Korlach, Tyson A Clark, Craig T Parker, Steven Huynh, Patrick S Chain, Sanaa Ahmed, Michelle Qiu Carter

**Affiliations:** 1 Produce Safety and Microbiology Research Unit, Western Regional Research Center, Agricultural Research Service, US Department of Agriculture, Albany, CA 94710, USA; 2 Pacific Biosciences, 1380 Willow Road, Menlo Park, CA 94025, USA; 3 Genome Sciences, Bioscience Division, Los Alamos National Laboratory, MS-M888, Los Alamos, NM 87545, USA

**Keywords:** Comparative genomics, Enterohemorrhagic *Escherichia coli*, Shiga toxin-producing *Escherichia coli*, DNA methylation, O145

## Abstract

**Background:**

Although serotype O157:H7 is the predominant enterohemorrhagic *Escherichia coli* (EHEC), outbreaks of non-O157 EHEC that cause severe foodborne illness, including hemolytic uremic syndrome have increased worldwide. In fact, non-O157 serotypes are now estimated to cause over half of all the Shiga toxin-producing *Escherichia coli* (STEC) cases, and outbreaks of non-O157 EHEC infections are frequently associated with serotypes O26, O45, O103, O111, O121, and O145. Currently, there are no complete genomes for O145 in public databases.

**Results:**

We determined the complete genome sequences of two O145 strains (EcO145), one linked to a US lettuce-associated outbreak (RM13514) and one to a Belgium ice-cream-associated outbreak (RM13516). Both strains contain one chromosome and two large plasmids, with genome sizes of 5,737,294 bp for RM13514 and 5,559,008 bp for RM13516. Comparative analysis of the two EcO145 genomes revealed a large core (5,173 genes) and a considerable amount of strain-specific genes. Additionally, the two EcO145 genomes display distinct chromosomal architecture, virulence gene profile, phylogenetic origin of Stx2a prophage, and methylation profile (methylome). Comparative analysis of EcO145 genomes to other completely sequenced STEC and other *E. coli* and *Shigella* genomes revealed that, unlike any other known non-O157 EHEC strain, EcO145 ascended from a common lineage with EcO157/EcO55. This evolutionary relationship was further supported by the pangenome analysis of the 10 EHEC str ains. Of the 4,192 EHEC core genes, EcO145 shares more genes with EcO157 than with the any other non-O157 EHEC strains.

**Conclusions:**

Our data provide evidence that EcO145 and EcO157 evolved from a common lineage, but ultimately each serotype evolves via a lineage-independent nature to EHEC by acquisition of the core set of EHEC virulence factors, including the genes encoding Shiga toxin and the large virulence plasmid. The large variation between the two EcO145 genomes suggests a distinctive evolutionary path between the two outbreak strains. The distinct methylome between the two EcO145 strains is likely due to the presence of a *Bsu*BI/*Pst*I methyltransferase gene cassette in the Stx2a prophage of the strain RM13514, suggesting a role of horizontal gene transfer-mediated epigenetic alteration in the evolution of individual EHEC strains.

## Background

Enterohemorrhagic *Escherichia coli* (EHEC) are a subset of Shiga toxin-producing *E. coli* (STEC) strains that cause severe foodborne-disease, including hemorrhagic colitis (HC) and hemolytic uremic syndrome (HUS). The classical characteristics of EHEC include the expression of Shiga toxin, production of attaching-and-effacing (A/E) lesions on epithelial cells, and possessing the large virulence plasmid [1]. *E. coli* O157:H7 (EcO157) is a prototype of EHEC and has been considered the most frequent cause of EHEC associated outbreaks [2-5]. However, it has become evident that non-O157 EHECs and STECs have emerged and are causing a large number of human infections worldwide. It is estimated that non-O157 STECs cause between 50-66% of all STEC infections in the United States [6-11]. For example, the recent large outbreak of *E. coli* O104:H4 (EcO104) in Europe caused 4,075 cases of STEC infection, 908 cases of HUS, and 50 deaths [12]. This STEC strain emerged from an enteroaggregative *E. coli* (EAEC) strain by acquiring genes encoding Shiga toxin [13]. Furthermore, a recent study suggested that up to 30% of patients who develop STEC-associated HUS will suffer long-term complications, including hypertension, neurologic symptoms, and decreased kidney function [14]. As a result, the World Health Organization (WHO) declared virulent non-O157 STECs a public health priority [15]. Currently, over 250 different STEC serotypes been described, and over 100 of those serotypes have been associated with human diarrheal disease [6,9,15]. The serotypes O26, O45, O103, O111, O121, and O145, also known as the "big six", are associated with human disease most frequently [9,16-18]. In fact, the US Department of Agriculture now requires testing for the presence of these STECs in all non-intact beef products [16,17].

There has been extensive research investigating the evolution of *E. coli*, a species that comprises commensal strains residing naturally in intestinal tracts of their mammalian hosts as well as pathogenic strains causing diverse intestinal and extraintestinal infections in humans and animals. Genome sequencing of the first EcO157 strain EDL933 and comparative analysis with the *E. coli* K-12 strain MG1655 revealed a considerable amount of genome plasticity [19-22]. For example, the genomes of EcO157 strain EDL933 and *E. coli* K-12 strain MG1655 differ in size by 1.0 Mb. EDL933 contains more than 1,000 additional genes compared to the MG1655, and many of these genes are located in varying size 'islands’ known as O-islands; similarly, strain MG1655 carries unique genes that are not found in EDL933 and these genes are located on various sized K-islands [21]. Many genetic determinants that encode the virulence of EDL933 are located in O-islands, including the Shiga toxin converting prophage and the locus of enterocyte effacement (LEE). Furthermore, EDL933 carries a large virulence plasmid that encodes for an enterohemolysin, a catalase, several proteins related to lipid A modification, and proteases [23,24]. Recently, complete genome sequences of more EHEC strains were determined, including two strains of EcO157 linked to the 2006 spinach-associated outbreak [25,26], and strains of O26, O103, and O111 linked to several sporadic outbreaks in Japan [22], which provided valuable information in understanding the evolution of EHEC strains. EHEC strains evolved from at least two separate lineages. EHEC/EPEC lineage 1 contains O157:H7 and its "progenitor" O55:H7. EcO157 is characterized by its ability to produce Shiga toxin, and inability to ferment sorbitol (SOR) and express β-glucuronidase activity (GUD). The emergence of EcO157 has been described by a stepwise model, in which EcO157 evolved in a series of steps from O55:H7 by acquiring a *stx2* gene, conversion to serotype O157, acquiring a *stx1* gene and changes associated with conversion to SOR^-^ and GUD^-^[27-29]. The EHEC/EPEC lineage 2 contains non-O157 serotypes O26, O103, and O111. Evolution of these EHEC strains has been proposed through a lineage-independent parallel mechanism, in which strains of various serotypes acquired virulence determinants independently [22]. Furthermore, it has been suggested that although different EHEC lineages vary in their virulence repertoire and in their global distribution [30-33], EHEC strains carry a core set of virulence factors [15,22,31].

To the date of our analysis, there are eight EHEC strains with complete genome sequences (fully closed genomes), including five EcO157 strains, and one strain each of EcO26, EcO103, EcO111, respectively [21,22,25,26,34,35]. The genomes of EHEC strains have been shown to be rich in prophages, integrated elements, and insertion sequences. This intrinsic feature corresponds to the presence of numerous identical sequences in the genome, some relatively long (>3,000 bp), which, with previous sequencing capabilities, posed traditionally a big challenge in genome assembly and gap closure. In this study, we determined the complete genome sequences of two highly virulent *E. coli* O145 (EcO145) strains linked to two separate outbreaks of EHEC infection in the US [36,37] and Europe [38,39] by compiling sequences generated by Roche 454, Illumina, and Pacific Biosciences (PacBio) sequencing platforms. We were able to finish two high quality (evidenced by average of >50X coverage) and fully closed EcO145 genomes quickly, using a strategy benefitting from long sequence reads (PacBio) and similar to a study reported previously [40]. We then performed comparative genomic analyses between the EcO145 strains and to other fully sequenced EHECs, STECs and other *E. coli*/*Shigella* strains available in public databases to gain insight into the genome and virulence evolution of EHEC.

## Results

### Genomic signature of EcO145 strain RM13514, a US lettuce-associated outbreak strain

**Figure 1:**
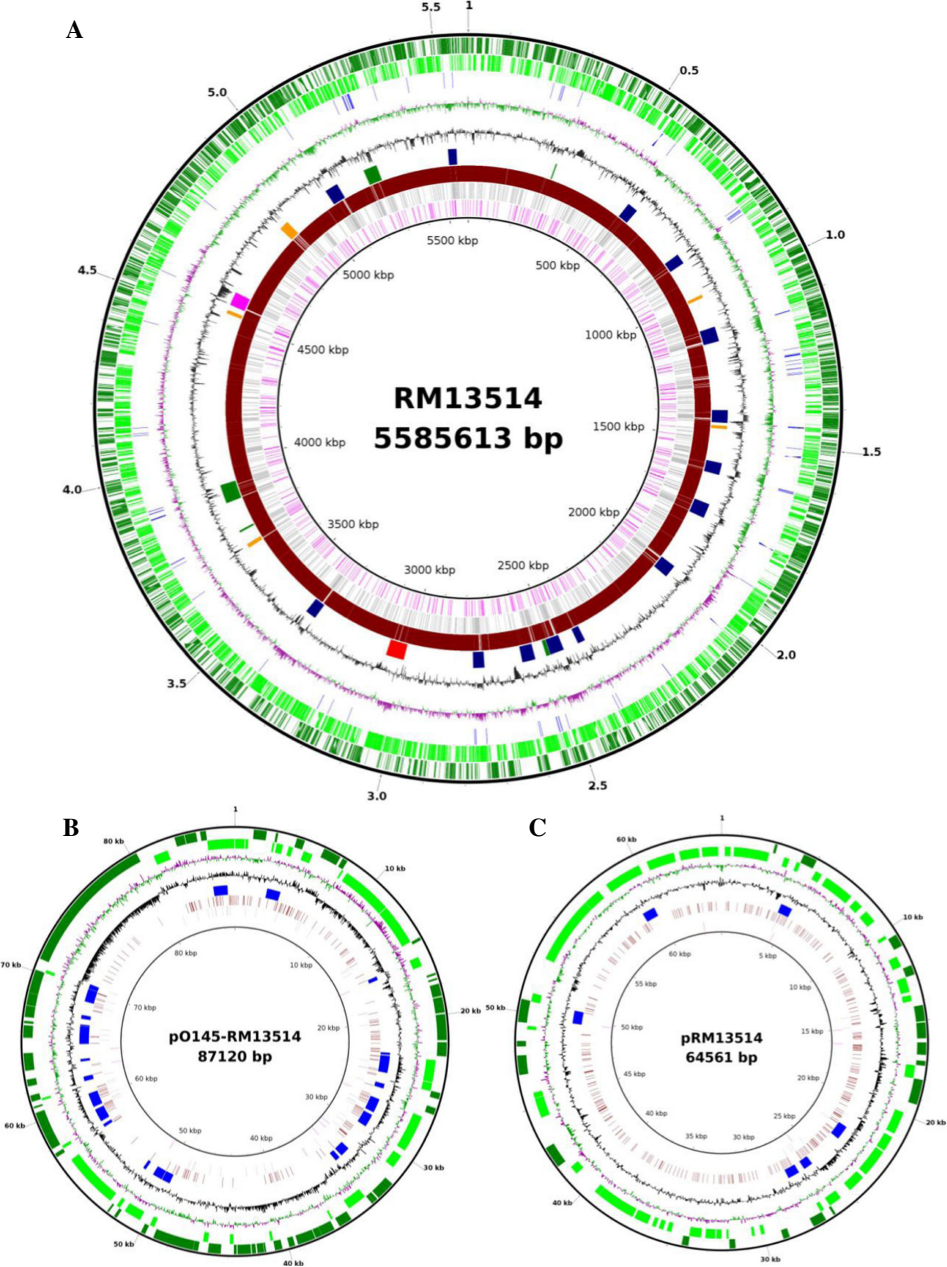
**Genome features of** ***Escherichia coli*** **O145:H28 strain RM13514.** Circular maps of the chromosome **(A)**, and the two plasmids, pO145-13514 **(B)** and pRM13514 **(C)**. For chromosome, from outer circle to inner circle, each represents positive strand CDS (1), negative strand CDS (2), insertion sequences (3), GC Skew (4), GC Content (5), Prophage/Integrated elements (Prophage–Navy, Prophage-like–Orange, Shiga toxin phage–Red, Integrated element–Green, LEE–pink) (6), GATC methylation sites (7), CTGCAG methylation sites (8), and DTGCAG methylation sites (9). For both plasmids, from outer circle to inner circle, each represents positive strand CDS (1), negative strand CDS (2), GC Skew (3), GC content (4), Insertion sequences (5), GATC methylation sites (6), CTGCAG methylation sites (7), and DTGCAG methylation sites (8). The circular maps were generated using BLAST Ring Image Generator (BRIG) software [41].

The genome of RM13514 is composed of a 5,585,613 bp chromosome and two large plasmids, pO145-13514 (87,120 bp) and pRM13514 (64,561 bp) (Figure 1). The chromosome consists of 5,613 coding DNA sequences (CDSs), 22 rRNA, and 104 tRNAs. Among the annotated CDSs, 73.8% have been assigned to at least one COG functional category. The backbone of the RM13514 chromosome is interrupted by numerous mobile elements, including prophage/prophage-like elements (20), integrated elements (7) or insertion sequences (91) (Table 1). The prophage that carries the genes (*stx2a*) encoding Shiga toxin in strain RM13514 is about 50 kb in size, and located adjacent to the *argW* locus. The LEE island is integrated at the *selC* locus in strain RM13514, analogous to EcO157, but differs from the other non-O157 EHEC strains (Table 1). Notably, the LEE island is absent in the O104:H4 German outbreak strain that has been shown by whole genome analysis to have evolved from an EAEC [12].

**Table 1:** **Genome characteristics of** ***Escherichia coli*** **O145:H28 and comparison with other genomes of STEC**

	***E. coli *** **O145:H28**		***E. coli *** **O157:H7**					***E. coli *** **O103 str. 12009**	***E. coli *** **O26 str. 11368**	***E. coli *** **O111 str. 11128**	***E. coli *** **O104 str. 2011C-3493**
	**RM13514**	**RM13516**	**EDL933**	**Sakai**	**EC4115**	**TW14359**	**Xuzhou21**				
**Chromosome**											
Size (bp)	5,585,613	5,402,276	5,528,445	5,498,450	5,572,075	5,528,136	5,386,223	5,449,314	5,697,240	5,371,077	5,273,097
%GC	50.7	50.7	50.4	50.5	50.5	50.5	50.5	50.7	50.7	50.6	50.7
CDSs	5,613	5,324	5,298	5,230	5,315	5,255	5,039	5,054	5,364	4,972	4,975
tRNA	104	98	98	105	110	106	93	98	101	107	94
rRNA	22	22	22	22	22	22	22	22	22	22	22
Prophages (Prophage-like elements)	15 (5)	10 (2)	9 (6)	13 (5)	10 (6)	10 (6)	8 (5)	11 (4)	13 (8)	15 (2)	6 (3)
*stx* genes	*stx2a*	*stx2a*	*stx1*+*2a*	*stx1+2a*	*stx2a +2c*	*stx2a+2c*	*stx1+2a*	*stx1 + 2a*	*stx1*	*stx1 + 2a*	*stx2a*
Number of IS	91	73	82	81	72	65	68	100	95	101	87
Number of IE	7	7	5	6	5	5	5	6	9	7	5
LEE-island integration locus	*selC*	*selC*	*selC*	*selC*	*selC*	*selC*	*selC*	*pheV*	*pheU*	*pheV*	N/A
**pEHEC-like plasmid**											
Size (bp)	87,120	98,066	92,077	92,721	94,644	94,601	92,728	75,546	85,167	77,690	N/A
GC (%)	47.6	49.7	47.6	47.6	47.9	47.9	47.6	49.1	47.5	50.0	N/A
CDSs	94	115	99	85	108	110	92	67	65	72	N/A
Number of IS	27	16	10	11	12	10	12	13	26	16	N/A
**Other plasmid(s)**											
Size (bp)	64,561	58,666	N/A	3,306	37,452	N/A	37,785	N/A	63,365/5,686/4,073	204,604/97,897/8,140/6,673	88,544/74,217/1,549
%GC	52.6	42.4	N/A	43.4	39.7	N/A	40.5	N/A	52.5/46.2/44.1	47.0/48.2/49.6/50.2	49.7/47.1/50.8
CDSs	69	73	N/A	3	54	N/A	52	N/A	81/6/3	222/121/10/10	94/80/1
Number of IS	6	0	N/A	0	0	N/A	0	N/A	1/0/0	14/2/0/0	18/3/0
**Total genome size (bp)**	5,737,294	5,559,008	5,620,522	5,594,477	5,704,171	5,622,737	5,516,736	5,524,860	5,855,531	5,766,081	5,437,407
**Total genome CDSs**	5,776	5,512	5,397	5,318	5,477	5,365	5,183	5,121	5,519	5,407	5,150

Plasmid pO145-13514 appears to be related to the plasmid pO157, as it carries several virulence genes that are also present on pO157, including the *hlyBCDA* operon, encoding the enterohemolysin and its secretion apparatus; *espP* gene, encoding a serine protease; *toxB* gene, encoding a homolog of large clostridial toxin ToxB; and genes encoding an adenine-specific methyltransferase as well as the enzymes related to lipid A biosynthesis/modification (a polysaccharide deacetylase, a glycosyltransferase, a metal-dependent hydrolase, and a lipid A biosynthesis protein ((KDO) 2-(lauroyl)-lipid IVA acyltransferase). However, pO145-13514 is also notably different from pO157, evidenced by the fact that this plasmid lacks *katP*, *flmABC*, as well as the operon encoding a general secretion system present on pO157 (*etpC*-*etpO*), but carries a v*agCD* toxin-antitoxin gene cassette and a *yebF*-like gene encoding a colicin immunity protein.

The second large plasmid in strain RM13514 (pRM13514) contains 69 CDSs. Unlike pO145-13514, this plasmid carries fewer IS elements (6 compared with 27 in pO145-13514), but with a higher GC content (52.6% compared with 47.6% of pO145-13514). The most striking feature of pRM13514 is the cluster of genes conferring multidrug resistance, including tetracycline (*tetA*), chloramphenicol/florfenicol (*bcr*), streptomycin (*strAB*), and sulfonamides (*sul1*). Additionally, the gene encoding dihydropteroate synthase (DHPS) is adjacent to *strAB*, conferring cells resistance to dapsone. The plasmid pRM13514 also carries several genes encoding proteins involved in DNA replication and transfer, such as *traG*, *traH*, and *traF*, however, the mobility of this plasmid remains to be determined.

### Comparative genomic analysis of EcO145

**Figure 2:**
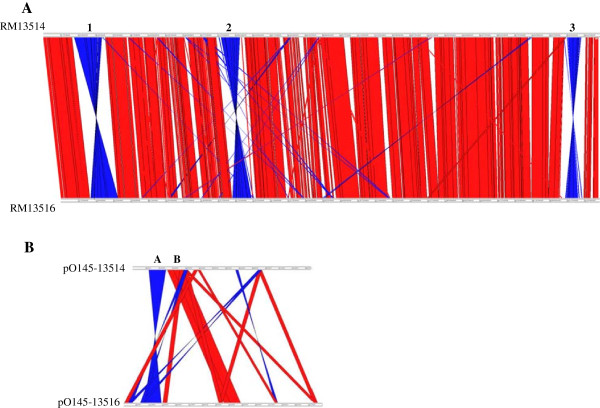
**Comparative analysis of** ***E. coli*** **O145:H28 genomes.** ACT alignment of the two *E. coli* O145:H28 chromosomes **(A)** and the two virulence plasmids, pO145-13514 and pO145-13516 **(B)** to demonstrate the conservation and the divergence between the genomes of the strains. Red color indicates the synteny between the two genomes, blue indicates the inversion, whereas white indicates no homologous sequences present between the two genomes. Regions 1, 2, and 3 on chromosome spanning the chromosome segments of 310,677-595,642, 1,789,072-1,980,931, and 5,269,949-5,398,918, respectively in strain RM13514. The first inversion in RM13514 is bordered by the mobile elements IE01 and P01, while the second inversion is bordered by P07 and P08, and the third inversion in RM13516 is bordered by IE06 and IE07. Regions A and B on pO145 represent a four gene cluster related to EHEC-hemolysin production and a four gene cluster related to lipid A modification, respectively.

Compared with the genome of RM13514, the genome of strain RM13516 (a Belgium ice-cream-associated outbreak strain), is about 180-kb smaller. It is composed of a 5,402,276 bp chromosome and two plasmids, pO145-13516 (98,066 bp) and pRM13516 (58,666 bp), encoding 5,324, 115, and 73 CDSs, respectively (Additional file 1: Figure S1 and Table 1). Similarly to RM13514, 73.9% of CDSs have been assigned to at least one COG category. The backbones of the two EcO145 chromosomes exhibit overall genomic synteny, with the exception of three major inversions (Figure 2A). The first inversion in RM13514 spans chromosome positions 315,760 to 596,570 (~280 kb), and appears to be unique to strain RM13514 since this region in strain RM13516 is syntenic with other EHEC strains including EcO157; similarly, the second inversion, from 1,789,020-1,982,030 (~190 kb) appears to also be inverted in strain RM13514. In contrast, the third inversion (~180 kb) appears to be unique to strain RM13516 (position: 5,060,402-5,242,158) as this region in strain RM13514 is syntenic with other EHEC strains (Figure 2A).

The RM13516 virulence plasmid (pO145-13516) shares limited genetic features with pO145-13514, namely the EHEC-enterohemolysin gene cluster (*hlyCABD*), the 4-gene cluster related to lipid A biosynthesis and modification, and the *psiBA* operon encoding plasmid SOS inhibition protein B and A (Figure 2B). Plasmid pO145-13516 carries a considerable number of genes that are absent from pO145-13514, such as the 11-gene cluster (*secCDEFGHIJKLM*) related to the type II secretion system; the 7-gene cluster related to IncF plasmid conjugal transfer apparatus (*finO*, and *traXIDTSG*); genes encoding RelB/RelE toxin/antitoxin system; genes encoding chromosome/plasmid partitioning protein ParA/ParB; and genes encoding the lesion bypass DNA polymerase V (*umuC*), lipoprotein TagA (*tagA*), and colanic acid biosynthesis acetyltransferase WcaB (*wcaB*).

Unlike pRM13514, pRM13516 does not carry any known drug resistance genes; rather, it is characterized by the two large gene clusters, encoding type IVb pilus (*pilL*-*M*, *pilN*-*V*) and type IV secretion system that is highly similar to the gene cluster encoding T-DNA transfer apparatus (*virD4*, *virB1-11*). Interestingly, the organizations of the two gene clusters were shuffled and re-arranged considerably in pRM13516, resulting in the insertion of genes *virB1*-*4*, *virB7*-*11*, and *virD4* between *pilM* and *pilN* genes (Additional file 1: Figure S1C).

Comparative analysis of the coding regions of the two EcO145 genomes revealed that they share 5,173 common genes, with 603 and 462 genes unique to strain RM13514 and RM13516, respectively. Although a large portion of strain-specific genes for either RM13514 or RM13516 are hypothetical or mobile element-related genes, strain RM13514 contains 53 unique genes with annotated functions, and many are related to metabolism or DNA replication and modification. Notably, there are eight methyltransferase encoding genes present only in RM13514, and, additionally, five are present in prophages (Additional file 2: Table S1). In contrast, among the 41 RM13516-specific genes, there were no methyltransferases; rather, many of the RM13516-specific genes encode functions in fatty acid biosynthesis, cell stress resistance, and DNA/protein secretions (Additional file 2: Table S1).

### EcO145 methylomes

The marked difference in the number of genes encoding methyl transferases between the two EcO145 strains led us to compare the global methylation profiles (methylomes) between the two strains. We took advantage of Pacific Biosciences’ (PacBio) single molecule [42], real-time (SMRT) sequencing technology’s capability to determine base modifications during sequencing to identify putative methylation sites across both genomes, and identified that both genomes had adenine methylated exclusively. A high percentage of the 5′-G**A**TC-3′ motif sites were detected to be adenine methylated in both genomes (Table 2 and Additional file 1: Figure S2, 97.8% and 98.9% for RM13514 and RM13516, respectively; modified base in bold, underlined base indicates methylation on the complementary DNA strand), suggesting a functional role of DNA adenine methylase (Dam) in both strains. In contrast, a distinct difference in adenine modification was observed for 5′-CTGC**A**G-3′ and 5′-DTGC**A**G-3′ motif sites between the two strains. In strain RM13514, 98.8% of the adenines were detected as methylated in the motif 5′-CTGC**A**G-3′ (total of 2,902 motifs), whereas in RM13516, none of the adenines in the 2,906 motifs were detected as methylated (Table 2 and Additional file 1: Figure S2). Similarly, in strain RM13514, about 3.9% of the sites of motif 5′-DTGC**A**G-3′ were detected to be methylated, whereas in strain RM13516 less than 0.05% of the sites were detected as methylated (Table 2 and Additional file 1: Figure S2). Methylation of adenine in 5′-CTGC**A**G-3′ in strain RM13514 is predicted to occur by the methylase of the type II restriction-modification (R-M) *Bsu*BI/*Pst*I system. Genes encoding the *Bsu*BI/*Pst*I R-M (ECRM13514_3159 and ECRM13514_3160) system are located in the Stx2a prophage. These two genes encode proteins that are extremely similar to enzymes shown biochemically to recognize CTGCAG (either methylate or cleave that sequence) previously [43]. The alignment of the protein encoded by ECRM13514_3160 with the DNA methylase M.EcoGIII is shown in Additional file 1: Figure S2C. The methylase M. EcoGIII, cloned from EcO104 strain C227-11, was shown to specifically methylate the adenine in the DNA motif CTGCAG [43]. The only difference is the foreshortening of this new gene, a phenomenon that has been observed in other methylases. The R gene of RM13514 is 100% identical to that of the strain C227-11, which was shown to be biochemically active [43]. Interestingly, this *BsuBI/PstI* R-M system is absent in strain RM13516, but conserved in the Stx2a prophage of the EcO104 strain 2011C-3943 and EcO103 strain 12009.

**Table 2:** **Methylation profiles of** ***E. coli*** **O145:H28 strains**

**Motif**	**Putative enzyme involved**	**Strain**	**Total motifs/genome**	**Total methylated (A) motif/genome**	**% of methylated motifs**
5′-G **A** TC-3′	DNA adenine methylase (Dam)	RM13514	44,248	43,268	97.8%
		RM13516	43,398	42,937	98.9%
5′-CTGC **A** G-3′	Modification methylase PstI	RM13514	2,902	2,868	98.8%
		RM13516	2,906	0	0.0%
5′-DTGC **A** G-3′	Unknown	RM13514	13,292	512	3.9%
		RM13516	12,824	6	0.0%
Unassigned	NA	RM13514	NA	126	NA
		RM13516	NA	191	NA

The motif 5′-DTGC**A**G-3′ is asymmetric, while 5′CTGC**A**G-3′ is a symmetric motif, and it is possible that methylation of adenine in the motif 5′-DTGC**A**G-3′ is due to non-specific activity of the *BsuBI/PstI* methylase, or catalyzed by an un-characterized, possibly type IIG methylase (Richard Roberts, personal communication).

### Phylogeny of EcO145

**Figure 3:**
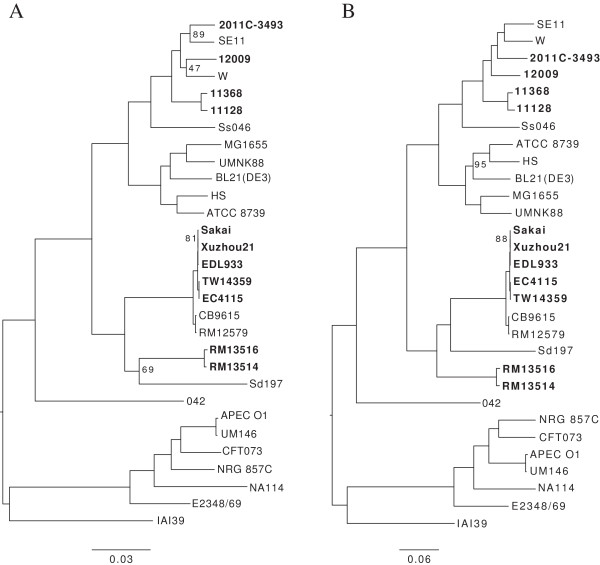
**Genome-wide phylogenetic analysis of 30 completely sequenced *E. coli*** **and** ***Shigella*** **strains. A**, maximum likelihood phylogenetic tree, based on the concatenated nucleotide sequences of 341 orthologous CDSs from each of the strains (Additional file 3: Dataset S1). The tree was constructed using RaxML with the JTT + GAMMA + Invariable sites model with 100,000 pseudoreplicates. The 341 CDS were selected from 345 CDS that were previously determined as being non-recombinogenic [22]. Four of 345 genes were removed because a subgroup of strains does not have these genes. **B**, maximum likelihood phylogenetic tree, constructed using genome-wide orthologous SNPs from 30 complete genomes. Pairwise comparisons of all genome sequences were carried out using NUCmer from the MUMer package [46] and highly similar regions (repeated sequences) were removed from the analysis. Orthologous SNPs are defined as SNPs present within the remaining alignments among all genomes. Only those SNPs present within the coding (CDS) regions were used for further phylogenetic analysis. The best substitution model (GTR + G) for the analysis was selected using ModelTest [47], and the tree was constructed using RAxML [48] with100,000 bootstrap replicates. The EHEC strains (EcO157: Sakai, Xuzhou 21, EDL933, EC4115 and TW14359; EcO145: RM13514 and RM13516; EcO26: 11368; EcO103: 12009; EcO111: 11128) and the STEC strain (EcO104: 2011C-3493) are in bold. Scale bar: number of substitutions per base. Only bootstrap value <= 95 are displayed.

The maximum-likelihood tree constructed using the concatenated nucleotide sequences of 341 orthologous CDSs (Additional file 3: Dataset S1) from 30 *E. coli* and *Shigella* strains suggests that EcO145 shares a common evolutionary lineage with O157:H7, O55:H7, and *S. dysenteriae*, whereas other non-O157 EHEC strains such as 12009 (O103), 11368 (O26), and 11128 (O111), along with the German outbreak STEC strain 2011C-3493 (O104), share a common evolutionary lineage with non-pathogenic *E. coli* strains, including strain W and SE11 (Figure 3A). As expected, the two EcO145 strains were grouped together. A similar phylogeny was observed for EcO145, EcO157, and other non-O157 EHECs when all the orthologous SNPs located in the coding regions of 30 genomes were used for tree construction (Figure 3B). In both trees *S. dysenteriae* was clustered together with EcO145, EcO157 and EcO55:H7, supporting the theory that *Escherichia coli* and *Shigella* spp. belong to the same species [44,45]. Slight differences in placement of a few strains were observed between the two trees, including *E. coli* strains SE11, W, NRG 857C, and CFT053, and *S. dysenteriae* strain Sd197. *S. dysenteriae* shares a common ancestor with EcO145 in the phylogenetic tree constructed using 341 CDSs, whereas in the genome-wide SNP-based tree, it is more closely related to EcO157 than EcO145 (Figure 3). *E. coli* strains SE11 and W are clustered together in the SNPs-based tree, but not in the CDSs-based tree. A similar shift was observed for strains NRG 857C and CFT073 (Figure 3). EcO145 appears to diverge from EcO157 prior to the separation of O157:H7 from the O55:H7 enteropathogenic *Escherichia coli* (EPEC) strain (Figure 3). Consistently, both EcO145 strains express β-glucuronidase activity, a trait that was conserved in EcO55 but lost in EcO157 due to mutations. Therefore, similar to other non-O157 EHEC strains including O26, O103, and O111, acquisition of the Shiga toxin encoding gene in EcO145 is lineage-independent.

### Comparative analysis of EHEC genomes

**Figure 4:**
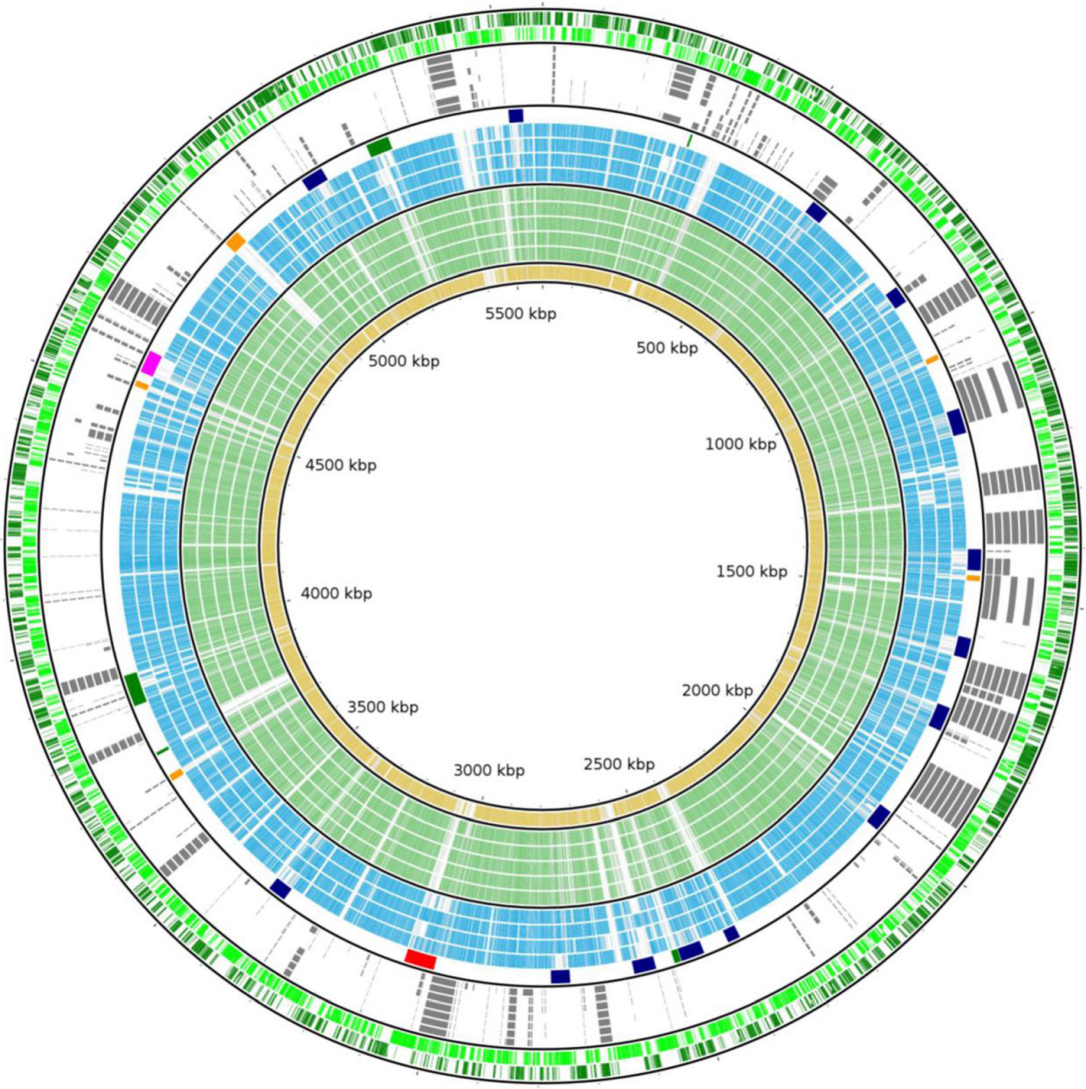
**Comparative analysis of** ***E. coli*** **O145:H28 with other EHEC strains.** BLAST comparison of complete EHEC chromosomes against EcO145 str. RM13514 chromosome, using BLASTP with a cutoff value of 75% identity. Genomes are ordered from inner to outer ring: (1) *E. coli* O145:H28 str. RM13516 (yellow); (2-6) *E. coli* O157:H7 strains EC4115, TW14359, Sakai, EDL933, and Xuzhou21(light green); (7-9) non-O157 STEC strains 11368 (O26:H11), 12009 (O103:H2), and 11128 (O111:H-) (blue); (10) *E. coli* O145:H28 str. RM13514 mobile elements [Prophage–Navy, Prophage-like–Orange, Shiga toxin phage–Red, Integrated element–Green, LEE – pink]; (11-16) O-islands present in O157 str. EDL933, O145 str. RM13514, O145 str. RM13516, O26 str. 11368 (14), O103 str. 12009, and O111 str. 11128 (grey); (17) RM13514 negative strand CDS (lime); and (18) RM13514 positive strand CDS (green). The circular plot was generated using BLAST Ring Image Generator (BRIG) software [41]. EHEC core genome genes and serotype unique genes compared to O145 strains are in Dataset S2, and list of O-islands present in non-O157 genomes are in Additional file 5: Dataset S3.

A pangenome analysis of the chromosomes of 10 EHEC strains identified a core of 4192 genes (Figure 4 and Additional file 4: Dataset S2). A large portion of EHEC core genes are conserved in EcO55 (3903 genes) and in the porcine UMNK88 strain (3805 genes). As expected, EcO145 shares more genes with EcO157 than with any other non-O157 EHEC strain. There are 210 genes only found in strains of EcO157 and EcO145; most are located in O-islands, and are associated with functions related to fatty acid synthesis, C5-branched dibasic acid metabolism, iron utilization, and type III secretion regulation (Additional file 4: Dataset S2 and Figure 4). There are 130 genes specific to both EcO145 and the other non-O157 EHEC strains (Additional file 4: Dataset S2), including genes related to phenylacetic acid degradation and glyoxylate, dicarboxylate, and fatty acid metabolism (Additional file 4: Dataset S2). BLASTP search of all EcO145 CDSs against other EHEC genomes revealed 138 genes that are specific to serotype O145:H28. Although a large portion of these genes encode hypothetical proteins, the search also revealed genes related to LPS biosynthesis, type I restriction system, adhesion/invasins, and CRISPR-associated proteins (Additional file 4: Dataset S2).

### Genomic islands and integrative sequences

Because of the close evolutionary relationship of EcO145 and EcO157, we examined the conservation of the 177 EcO157 O-islands in genomes of non-O157 EHECs and the STEC O104 (Additional file 5: Dataset S3). The extent of the conservation in either of the EcO145 strains is greater than any of the non-O157 EHEC strains (O26, 42.9%; O103, 43.5%; O111, 42.3%) or the O104:H4 strain (36.7%). Part or all of 136 (76.8%) and 130 (73.4%) EcO157 O-islands were detected in strains RM13514 and RM13516, respectively. The large genomic islands that appear to separate the EHEC/EPEC linage I from the EHEC/EPEC lineage II include OI-28, OI-47, and OI-138. Both OI-28 and OI-138 are fully conserved in both EcO145 strains; whereas the OI-47 is partially retained in strain RM13514 (26.6%), but fully conserved in strain RM13516 (Additional file 1: Figure S3). The large islands OI-43, OI-48, OI-115, OI-122, and OI-148 are conserved in all 10 EHEC strains. OI-43 and OI-48 are highly similar, and both known as tellurite resistance islands (Additional file 1: Figure S4). Besides the tellurite resistance genes, both islands carry the genes encoding proteins necessary to synthesize urease, an enzyme that has been suggested to play a role in cell acid resistance in the host GI tract [49]. Similar to EDL933, RM13514 carries two tellurite resistance islands. In contrast, RM13516 carries only one tellurite resistance island (OI-48), similar to the other non-O157 EHEC and STEC strain O104 (Additional file 1: Figure S4).

The pathogenicity island LEE is a molecular characteristic of EHEC strains responsible for the formation of A/E lesions on epithelial cells. The LEE island (OI-148) for both EcO145 strains are integrated at tRNA (*selC*), analogous to EcO157 strains, and are also similar in size (RM13516, 47,948 bp; RM13514, 46,793 bp) to EcO157 (43,323-43,653 bp). The LEE islands in other non-O157 EHEC strains are generally larger in size (54,269-87,535 bp) and integrated at either the *pheU* or *pheV* locus (Additional file 1: Figure S5A; Additional file 6: Dataset S4). Further analysis of all LEE genes reveals a core of 38 genes that are conserved in all 10 EHEC genomes (Additional file 1: Figure S5B). The EcO145 LEE islands are highly similar to that of EcO157 (83% sequence identity across nearly 39-kb of the LEE island); whereas the other non-O157 strains have more complex LEE accessory regions. Phylogenetic analysis of the LEE reveals a similar phylogeny to that derived from whole genome-based comparisons (Additional file 1: Figure S5C). Both OI-115 and OI-122 are related to T3SS and are partially conserved in EcO145. The OI-115 diverged largely in other non-O157 EHEC strains (Additional file 1: Figure S6A); whereas the OI-122 in O145 is more similar to O103 than O157 including the integrated site (the *pheV* locus in strain EDL933, but the *pheU* locus in O145 and other non-O157 genomes) (Additional file 1: Figure S6B).

### Prophages and ISs

RM13514 and RM13516 have 20 and 12 prophage/prophage-like elements, respectively, similar to the range found in other STEC strains (Table 1). In both O145 strains, lambda or lambda-like phages are predominant (Additional file 2: Table S2). Except for EcO26, all eight EHEC strains and the STEC O104 strain contain a Stx2a prophage (Additional file 1: Figure S7A; Additional file 7: Dataset S5). Phylogenetic analysis of the Stx2a prophages suggests that, unlike the EcO157 strains, the Stx2a prophages of the two EcO145 are distantly related (Additional file 1: Figure S7B). The Podoviridae family Stx2a prophage in RM13514 is highly similar to those of the EcO103 strain 12009 and EcO104 strain 2011C-3493, whereas the lambda-like Stx2a prophage in RM13516 is closely related to that in EcO111. Further examination of the integration sites in EcO145 reveals a total of 32 putative sites; five are unique to EcO145, and 20 and 23 are shared with EcO157 and the other non-O157 STEC strains, respectively (Additional file 2: Table S3). Those integration sites appear to be unevenly distributed across the chromosomes in any of the STEC genomes (Additional file 1: Figure S8).

We detected 124 and 89 ISs in strains RM13514 and RM13516, respectively (Additional file 2: Table S4). The number of IS in RM13516 is similar to that in EcO157, whereas the number of IS in RM13514 is similar to that in other non-O157 STEC genomes. In both EcO145 strains, *IS629* appeared to be the most prevalent IS, followed by the *IS600* and the *ISEc8* (Additional file 2: Table S4). In fact, *IS629* appeared also to be the predominant IS element in all 10 EHEC strains, supporting its critical role in the evolution in EHEC [50-52].

### Plasmids

EHEC strains differ significantly in the total number and composition of plasmids (Table 1). The EHEC virulence plasmids display large variations in gene content and gene organization, indicating a distinct evolutionary history for each plasmid. Conservation of genes related to enterohemolysin and lipid A modification suggests they are part of the EHEC core virulence factors (Additional file 1: Figure S9). Alignment of plasmid sequences reveals that the five pO157s form three closely related groups, whereas other plasmids including both pO145-13514 and pO145-13516 diverge significantly. Further analysis of pO145-13514 reveals several segments related to the large plasmids of EcO26, including the 29-kb segment (57,904-87,120) containing genes *toxB*, *traG*, *traB*, and *repA* with a 98.5% identity to pO26-vir (GenBank accession no: FJ386569) and the 27-Kb DNA segment (33,181-60,611) containing genes *espP*, *nikB*, and *psiAB*, that was aligned perfectly with the plasmid pO26-CRL (GenBank accession no: GQ259888). The presence of IS elements or transposons at the borders of each DNA segment suggests a "mix and match" evolution path of the pO145-13514.

The multidrug resistance genes in the plasmid pRM13514 are located on a 21-kb DNA segment (5,017-26,289) that is also present on plasmids of *E. coli* (e.g. pUMNK88 and peH4H), *Salmonella (*e.g. pSH163_135, pSN254 and pSD-174*)*, and *Providencia stuartii* (pMR0211) (Additional file 1: Figure S10A). Interestingly, this large DNA segment is also present on a genomic island in *S. Typhimurium* (AB571791). Similarly, the 22-kb DNA fragment (27,534-49,368) of pRM13514 carrying genes *repA*, *clpP*, *dsbA,* etc. is also found in plasmids pTC2, pP91278, pNDM-KN isolated from *Providencia stuartii*, *Photobacterium damselae*, and *Klebsiella pneumonia* (Additional file 1: Figure S10A). pRM13516 does not appear to be related to any previously reported EHEC or STEC plasmids, rather, there is a large DNA segment containing type IVb pilus genes and *virB1*-*virB11* that are also present on *Escherichia coli* plasmids pChi7122-3 and pR721 and *Salmonella* plasmid pSH146-65 (Additional file 1: Figure S10B).

## Discussion

The rapid development of next generation sequencing technologies enables us to obtain the bacterial draft genomes quickly, however, it remains challenging to fully close a genome. This is particularly true for genomes of STEC due to the prevalence of mobile elements. We used second-generation sequence technology (Roche 454 and Illumina) to produce draft genomes of the EcO145 strains corresponding to 115 to 247 contigs that are difficult to close because of the common repetitive sequences. We then made use of error-corrected long reads (ranging from 547 bp to 10,796 bp) provided by PacBio sequence technology, which facilitated genome closure by spanning identical sequence with unique flanking regions for placement. The alignment of high coverage short reads along with an adequate number of informative long reads provides an extremely effective strategy for efficient closing and finishing of genomes containing multiple long identical sequences, regardless of size. To our knowledge, this is the first report on the complete genome sequence of EcO145, one of the "big six" non-O157 EHEC serotypes. The genomic information obtained in this study reveals the genomic diversity in EHEC, and contributes significantly to our understanding of genome and virulence evolution of EHEC strains.

Whole genome-based phylogenetic analysis reveals that EcO145 evolved from a common ancestor with EcO157, likely from an EPEC strain. It appears that the EcO145 diverged as a sub-lineage prior to the separation of EcO157 from the "progenitor" EcO55 EPEC strain, followed by acquisition of a Shiga-toxin converting prophage. This speculation is further supported by the observation that both EcO145 strains display GUD activity. Comparative genomics analyses of EcO145 with EcO55 and other EHEC strains reveals that EcO145 and EcO55 share nearly the same, or more, core genes than the number of core genes EcO145 share with other non-O157 EHEC strains. Furthermore, EcO145 and EcO157 share a larger core set of genes than the core of EcO145 and any other non-O157 EHEC strains. Examining the EcO157-specific genomic islands (O-islands) in EcO145 and the other non-O157 EHEC genomes also supports the common lineage of EcO145 with EcO157. EcO145 strains contain at least 30% more EcO157 O-islands than do any of the other non-O157 EHEC strains, including the large O-islands. Among four additional O-islands that were categorized as unique to EcO157 and the "progenitor" EcO55 EPEC genomes [3], three of these (OI-1, OI-47, and OI-141) are conserved in EcO145 genomes, but none of them were identified in other non-O157 EHEC genomes. Both LEE islands in EcO145 and EcO157 were integrated at the *selC* locus, whereas the LEE islands in the other non-O157 EHEC strains were integrated at the *pheV* or *pheU* locus [22,32]. Although all LEE islands share a core set of genes [19,33,53], EcO145 and EcO157 strains have a similar LEE accessory region (the up or downstream genes of the LEE island outside the defined core region), compared with other non-O157 strains. The O-island 140 is a nine-gene cluster related to iron acquisition, and in EcO145, it is inserted into the acid fitness island, analogous to EcO157, EcO55 and *S. dysenteriae*[54]. In contrast, none of the other non-O157 EHEC strains carry this island. These common genetic determinants as well as the gene organization patterns between EcO145 and EcO157 support their common evolutionary history, which serves possibly as the molecular basis for the common phenotypes shared by these two major EHEC serotypes. In fact, a recent study by CDC of the epidemiological features of STEC infection in the US found EcO157 (43%) and EcO145 (28%) have higher hospitalization rates than EcO26 (10%), EcO103 (11%), or EcO111 (14%) [55].

It has previously been shown that some non-O157 EHEC strains (O26, O103, and O111) arose from a different lineage (EHEC2/EPEC2) compared to EcO157 strains (EHEC1/EPEC1) via parallel evolution [22,30,32,33]. Comparative analysis of EcO145 with the other non-O157 EHEC strains reveals a total of 102 genes that are unique to EcO145 and non-O157 EHEC strains, including 18 genes related to degradation of phenylacetate, a common aromatic compound in the intestinal tracts of animals and humans, and 19 genes related to glyoxylate, dicarboxylate, and fatty acid metabolism. In EcO157 strains, we found the phenylacetate degradation gene cluster has been replaced by OI-67 (encodes three hypothetical proteins), whereas the 19-gene cluster related to glyoxylate, dicarboxylate, and fatty acid metabolism has been replaced by the OI-122, encoding accessory proteins of T3SS. Acquisition of OI-122 appears to be lineage-independent since in both EcO145 and the other non-O157 EHEC strains, OI-122 is integrated at the *pheU* locus, whereas in EcO157, the OI-122 is at the *pheV* locus. Additionally, both EcO145 and other non-O157 EHEC strains carry an eight gene cluster related to aspartate metabolism, which is absent in EcO157; similarly, both EcO157 and the other non-O157 EHEC strains carry the *frl* operon, which is absent in EcO145. Further examination of these gene clusters in EcO55 reveals that, similar to EcO145, it contains the 19-gene cluster related to fatty acid metabolism; similarly to EcO157, EcO55 lacks both the phenylacetate degradation genes and the aspartate metabolism genes. Therefore, it appears that the elimination of genes related to glyoxylate, dicarboxylate, and fatty acid metabolism in EcO157 occurred after separation of EcO157 and EcO55 lineages, whereas elimination of phenylacetate degradation genes and the aspartate metabolism genes occurred before the divergence of EcO157 from EcO55. Loss of the *frl* operon appears to be specific to EcO145, suggesting a role of gene loss in evolution of EHEC strains.

Mobile elements are known to play a key role in driving genome and virulence evolution of EHEC. A total of 24 different types of prophage were identified in 10 EHEC genomes, of which lambda-like phages are the most prevalent. Among the 10 EHEC strains examined, nine carry Stx2a prophages; EcO26 carries a Stx1 prophage. Both prophages and integrative elements are important sources of genes encoding T3SS effectors and other virulence-related proteins. Overall there are about 43-51 genes encoding T3SS effectors in the 10 EHEC strains, and the variation is largely due to the gene encoding effector NleG, which ranges from 6 to 16 copies in the genome (Additional file 2: Table S5). Additionally, the EcO145 strains do not carry the prophage encoding the EspW effector, which is present in O157 and the other non-O157 strains, whereas all non-O157 EHEC strains are missing the prophage-encoded NleD effector. However, only the EcO145 strains have functional copies of the prophage encoding EspV effector. It remains unclear how such variation impacts the virulence of EHEC strains.

The two EcO145 strains evolved to EHEC strains independently through gene acquisitions/gene loss, natural mutations, and genomic rearrangements. The chromosome of US lettuce-associated outbreak strain RM13514 is about 183 kb larger than that of the Belgium ice-cream associated outbreak strain RM13516, which relates mainly to the difference in prophage/prophage-like elements. Strain RM13514 carries eight prophage/prophage-like elements not present in RM13516, corresponding to more strain-specific genes in RM13514 than in RM13516. The Stx2a-prophage in the US outbreak strain (RM13514) belongs to the Podoviridae family, whereas the Stx2a-prophage in the Belgium outbreak strain (RM13516) is a lambda-like phage. Although genes on the LEE island (OI-148), as well as those on the T3SS-related islands (OI-122 and OI-155), are highly conserved between the two strains, nonsense mutations have been observed in several putative virulence genes in RM13514, implying loss of functions in the US outbreak strain. The pO145-RM13514 lacks the gene cluster encoding the type II secretion systems, whereas the pO145-RM13516 lacks the gene encoding the large clostridial toxin ToxB. Strain RM13514 evolved to be resistant to several common antibiotics including sulfonamides, streptomycin, tetracycline and chloramphenicol due to the acquisition of plasmid pRM13514. In contrast, the Belgium outbreak strain is susceptible to all the above antibiotics. Rather, it carries a second plasmid (pRM13516) encoding a type IV secretion system as well as a DNA conjugal transfer apparatus (type IVb pilus), suggesting a proficiency in DNA transfer and producing extracellular products. This independent acquisition of genes mediated by various mobile elements has also been reported in EcO157 and EcO55, leading to a variety of genomically related strains with distinct bacteriophage collections [29].

We observed distinct methylation profiles between the two EcO145 strains. Although both strains exhibited Dam methylation (adenine in the 5′-G**A**TC-3′ motif), only RM13514 exhibited adenine methylation at 5′-CTGC**A**G-3′ and 5′-DTGC**A**G-3′ motif sites. The 5′-CTGC**A**G-3′ motif would likely be recognized by the *Bsu*BI/*Pst*I type II restriction-modification (R-M) system (ECRM13514_3159 and ECRM13514_3160) located in the Stx2a prophage. DNA methylation in bacteria has been shown to play a role in replication, gene expression and virulence [43,56,57], as well as modulating phase variation of *agn43* in *E. coli*[58], phase variation of Pap pilus in *E. coli*[59], and control of O-antigen chain length in *Salmonella enterica*[60]. However, the implication of such methylation differences in these two O145 strains with respect to bacterial virulence and fitness remains to be determined.

## Conclusions

Our study is the first report on two complete EcO145 genomes. The genomic information obtained in this study promotes not only the identification of EcO145-specific genes, but also the recognition of EHEC core genes, which would facilitate the detection of STEC in food. Our whole-genome based phylogeny analysis demonstrated that O145 and O157:H7 strains ascended from the same EHEC1/EPEC1 lineage along with O55:H7 EPEC strains. While these strains shared a common EPEC ancestor, O145 strains formed a sublineage prior to acquiring the Shiga toxin-converting prophage(s). Once in the sublineage, similar to other non-O157 EHEC strains, O145 strains independently attained numerous virulence factors including Stx2a prophage and the EHEC plasmid. Additionally, our study also shows these mobile genetic elements not only contribute to gene content of EHEC strains, but also impact the epigenetics of the individual EHEC strain.

## Methods

### Bacterial strains

Strain RM13514 is a clinical isolate related to the 2010 romaine lettuce-associated outbreak in US [36,37]. Strain RM13516 is a clinical isolate linked to an outbreak of co-infection by EcO145 and EcO26 associated with consumption of ice cream in Belgium in October 2007 [38,39]. Both clinical strains were taken as part of standard care. No ethical approval was required for their use.

### Genome sequencing

Bacterial DNA was extracted from the stationary phase cultures grown in LB broth as previously described [61] with slight modification. Briefly, cells were lysed with SDS followed by sequential treatment with RNase A (1.0 mg/ml for 24 h) and proteinase K. The DNA was first precipitated in a sodium acetate/ethanol solution, and then purified by phenol/chloroform extraction, followed by the final ethanol precipitation. The purified DNA was re-suspended in Qiagen Buffer EB (QIAGEN) for genome sequencing.

For Roche 454 pyrosequencing, libraries were prepared for whole genome sequencing (WGS) and 8-kb insert paired-end (PE) sequencing according to the manufacturer’s protocol. Samples were barcoded and sequenced on a FLX Genome Sequencer (Roche) using the GS FLX Titanium system. A total of 353,416 WGS reads/337,391 PE reads and 249,287 WGS/54,954 PE reads were generated for RM13514 and RM13516, respectively. Illumina library preparation and sequencing were run (101 bp PE) at Ambry Genetics (Aliso Viejo, CA) on a HiSeq2000 sequencer. A total of 70,096,726 PE reads and 59,857,480 PE reads were generated for RM13514 and RM13516, respectively. PacBio libraries for continuous long read (CLR) and circular consensus sequence (CCS) reads were prepared according to the manufacturer’s protocols. PacBio SMRT sequencing was carried out on a PacBio RS instrument using C2 chemistry. A total of 297,437 CCS reads and 168,165 CLR reads, and 360,848 CCS reads and 134,983 CLR reads were generated for RM13514 and RM13516, respectively.

### Genome assembly and gap closure

The initial assembly was performed as previously described with modifications [62]. Briefly, 454 WGS and PE reads were assembled using Newbler (v2.3), and contigs broken into 2-kb overlapping fragments. Illumina PE reads were assembled using VELVET (v1.0.13), and contigs broken into 1.5-kb overlapping fragments. Polisher software was then run to compare the quality of the 454 and Illumina assemblies and proofread the consensus sequences. Finally, GapResolution and dupFinisher programs [62,63] were used to close gaps and correct mis-assemblies to generate an initial draft assembly, which contained 14 scaffolds composed of 247 contigs, and 12 scaffolds composed of 115 contigs for RM13514 and RM13516, respectively.

Optical maps for both strains were generated using the Argus optical mapping system (OpGen; Gaithersburg, MD), and the correct contig order and any mis-assemblies were determined. We initially closed gaps by primer walking via PCR and Sanger sequencing the amplified region, however, due to the complexity of numerous repeat regions, this strategy was very tedious and difficult. We then used PacBio long reads to close remaining gaps in the repeat regions. First, filtered PacBio CLRs were error-corrected with PacBio CCS reads utilizing the Celera assembler (v7.0) software and the PacBioToCA script [40]. Error-corrected PacBio CLRs were then aligned to the contigs using Geneious (v5.1) software [64], and the remaining gaps were manually closed *in silico* utilizing the Geneious software.

### Genome annotation

The completed genome sequences were submitted to Rapid Annotation using Subsystem Technology (RAST; rast.nmpdr.org) for the initial annotation [65], and then manually verified and corrected. The complete genome sequences are available at GenBank under the accession numbers: RM13514 chromosome (CP006027), pO145-13514 (CP006028), pRM13514 (CP006029), RM13516 chromosome (CP006262), pO145-13516 (CP006263), and pRM13516 (CP006264).

### Detection of DNA methylation

Detection of DNA methylation was carried out as previously described [42]. Briefly, PacBio CLR and CCS reads were mapped to the corresponding reference genomes using the Basic Local Alignment with Successive Refinement (BLASR; https://github.com/PacificBiosciences/blasr). Polymerase dynamics were measured and aligned for each base in the corresponding reference sequence as previously described [66,67] using the PacBio SMRTAnalysis pipeline ( https://github.com/PacificBiosciences/SMRT-Analysis/wiki/SMRT-Pipe-Reference-Guide-v2.0). Each modified base position was determined using PacBio SMRTPortal analysis (v1.3.1).

### Identification of prophage and integrated element

Prophage and prophage-like elements were analyzed with Prophage Finder Web server (bioinformatics.uwp.edu/~phage/ProphageFinder.php) [68] and PHAST Web server (phast.wishartlab.com) [69] for initial identification. Integrated elements were analyzed with the server MobilomeFINDER (db-mml.sjtu.edu.cn/MobilomeFINDER/) for initial identification [70]. Each of the identified prophages, prophage-like elements, and integrated elements were then examined manually for accuracy of the predication. Integrases not associated with any nearby identified element regions were manually assessed for the presence of a prophage, prophage-like element or integrated element.

### Whole genome-based phylogenetic analysis

Genomes used in the analysis were downloaded from GenBank, including eight EHEC strains (EcO103 str. 12009 [NC_013353], EcO26 str. 11368 [NC_013361], EcO111 str. 11128 [NC_013364], EcO157 str. EC4115 [NC_011353], EcO157 str. TW14359 [NC_013008], EcO157 str. Sakai [NC_002695], EcO157 str. EDL933 [NC_002655], EcO157 str. Xuzhou21 [NC_017906]), the German outbreak strain *E. coli* O104 str. 2011C-3493 [NC_018658], 19 other *E. coli*/*Shigella* strains (*E. coli* W [NC_017635], *E. coli* SE11 [NC_011415], *E. coli* ATCC 8739 [NC_010468], *E. coli* HS [NC_009800], *E. coli* BL21(DE3) [NC_012971], *E. coli* str. K12 subst. MG1655 [NC_000913], *E. coli* UMNK88 [NC_017641], *E. coli* O55 str. CB9615 [NC_013941], *E. coli* O55 str. RM12579 [NC_017656], *E. coli* 042 [NC_017626], *E. coli* IAI39 [NC_011750], *E. coli* O127 str. E2348/69 [NC_011601], *E. coli* NA114 [NC_017644], *E. coli* O83 str. NRG 857C [NC_017634], *E. coli* CFT073 [NC_004431], *E. coli* APEC O1 [NC_008563], *E. coli* UM146 [NC_017632], *Shigella sonnei* Ss046 [NC_007384], and *S. dysenteriae* Sd197 [NC_007606]), and the two EcO145 genomes sequenced in this study (RM13514 and RM13516).

Whole genome-based phylogeny was first constructed using 345 *E. coli* CDS that were identified previously with a low probability of recombination [22]. A total of 341 genes were conserved in all 30 genomes (Additional file 3: Dataset S1), thus the nucleotide sequences of these 341 genes from each genome were concatenated together and aligned using multiple sequence alignment program, MAFFT [71]. A maximum likelihood (ML)-based phylogenetic tree was constructed using RAxML program [48] with the JTT + GAMMA + Invariable sites model, based on model selection by ProtTest [72], and the reliability was assessed by bootstrapping 100,000 pseudoreplicates. We further examined consistency of this tree with one generated from whole genome orthologous SNPs (SNPs that were present in non-repeat regions of the genomes). These SNPs were identified from each genome relative to the sequence of RM13514, using NUCmer from the MUMer package [46] for pairwise comparisons of all genome sequences. SNPs present only in the coding (CDS) regions of the genomes were used for phylogenetic analysis. The best substitution model (GTR + G) for the analysis was selected by using ModelTest [47]. The resulting all CDS SNP tree was constructed using RAxML [48] with 100,000 bootstrap replicates.

### Genome alignment using Artemis Comparison Tool (ACT)

Either the chromosome or the plasmid sequences of EcO145 strains (FASTA format) were BLASTed against each other using the WebACT with default settings [73], and the two O145 genomes were aligned using ACT (v10.0.1) with the default settings [74].

### Comparative analysis of EHEC genomes

A core genome of the ten complete EHEC genomes was generated by creating a reference database of all the protein sequences present in RM13514, and then using the BLASTP program in the Geneious to compare all the protein sequences of nine EHECs (five EcO157 genomes, one of each EcO145 genome (RM13516), EcO111 genome, EcO103 genome, and EcO26 genome). The process was then repeated with each of the EHECs serving as reference protein database, and protein sequences that were present in all the EHECs with ≥ 75% identity across ≥ 75% of the sequence were considered a core sequence. Protein sequences that had <75% identity in all the other EHECs were considered unique for that strain. Unique CDSs for RM13514 and RM13516 were then compared against the NCBI database for presence in other *E. coli* strains. To determine the conservation of the EHEC core genome in other *E. coli* strains, a protein sequence database of each of the 19 *E. coli/Shigella* strains as described above was generated. The EHEC core genome was then compared to each database using BLASTP. Comparative analysis of the EcO145 strains was performed by searching all the proteins of the each O145 strain against the database containing all proteins of the both EcO145 strains by BLASTP. Protein sequences present in both strains with ≥90% identity were considered the O145 core genome, whereas proteins with sequences ≤90% identity were considered unique or accessory CDSs.

### MAFFT alignment

The nucleotide sequences of the LEE or plasmids were aligned using the MAFFT program, and ML-based phylogenetic trees were built using the RAxML programs with the previous described methods and parameters. The plasmid nucleotide sequences were analyzed for re-arrangements using progressive Mauve software to generate an alignment [75]. The nucleotide sequences for the virulence plasmid were obtained from GenBank under the following accession numbers: EC4115 pO157 (NC_011350), EDL933 pO157 (NC_007414), O26 pO26-1 (NC_013369), O103 pO103 (NC_013354), O111 pO111-3 (NC_013366), Sakai pO157 (NC_002128), TW14359 pO157 (NC_013010), Xuzhou21 pO157 (NC_017907). The nucleotide sequence for either pRM13514 or pRM13516 were used for a BLAST search on the NCBI website, and the nucleotide sequences for up to 10 related plasmids were obtained and used for analysis (pSH146_65 [JN983044], pR721 [AP002527], pChi7122-3 [FR851304], pAM04528 [FJ621587], pAR060302 [FJ621588], peH4H [FJ621586], pP91278 [AB277724], pSD_174 [JF267651], pSH111_166 [JN983043], pSH163_135 [JN983045], pSH696_135 [JN983048], pTC2 [JQ924049], and pUMNK88 [HQ023862]).

## Availability of supporting data

The complete genome sequences of both strains were deposited in GenBank database with the following links: http://www.ncbi.nlm.nih.gov/bioproject/?term=RM13514; http://www.ncbi.nlm.nih.gov/bioproject/?term=RM13516; The sequence data will become public available when this manuscript is accepted for publication. All the other supporting data are included as additional files.

## Abbreviations

EHEC: Enterohemorrhagic *Escherichia coli*; SNP: Single nucleotide polymorphism; STEC: Shiga toxin-producing *Escherichia coli*; HC: Hemorrhagic colitis; HUS: Hemolytic uremic syndrome; EcO157: *Escherichia coli* O157:H7; EcO104: *Escherichia coli* O104:H4; EcO145: *Escherichia coli* O145:H28; WHO: World Health Organization; LEE: Locus of enterocyte effacement; SOR: Ferments sorbitol; GUD: β-glucuronidase activity; CDS: Coding DNA sequence; ACT: Artemis Comparison Tool; BLASR: Basic Local Alignment with Successive Refinement; DHPS: Dihydropteroate synthase; SMRT: Single molecule, real time; Dam: DNA adenine methylase; R-M: Restriction-modification; RTX: Repeats in toxin; T3SS: Type III secretion system; EPEC: Enteropathogenic *Escherichia coli*; CLR: Continuous long reads; CCS: Circular consensus sequence; RAST: Rapid Annotation using Subsystem Technology.

## Competing interests

JK and TAC are full-time employees at Pacific Biosciences, a company commercializing single molecule, real-time sequencing technologies.

## Authors’ contributions

KKC contributed to experiment design, data gathering, data analysis, data interpretation and writing of the manuscript. REM contributed in experiment design and revision of the manuscript. JWL, TAC, SH contributed in data gathering, and SA contributed in data analysis. JK, CTP, PSC contributed in data analysis, data interpretation and revision of the manuscript. MQC supervised this study, contributed to experiment design, data analysis, data interpretation and writing of the manuscript. All authors read and approved the final manuscript.

## Supplementary Material

Additional file 1: Figure S1 Genome features of *Escherichia coli* O145:H28 strain RM13516; **Figure S2.** Detection of DNA methylation; **Figure S3.** Gene organization of the three large O-islands (OI) conserved between EcO145 and EcO157; **Figure S4.** Gene organization and content of the tellurite resistance islands; **Figure S5.** Gene organization and phylogenetic analysis of LEE island; **Figure S6.** Gene organization of the two large O-islands encoding T3SS-related proteins; **Figure S7.** Gene organization and phylogenetic analysis of Stx2a prophage; **Figure S8.** The chromosomal distribution of STEC major mobile elements; **Figure S9.** MAFFT and Mauve alignment of EHEC virulence plasmids; and **Figure S10.** MAFFT and Mauve alignment of EcO145 secondary plasmids. Click here for file

Additional file 2: Table S1 EcO145 strain-specific genes and their functional categories; **Table S2.***E. coli* O145:H28 prophage/prophage-like elements and integrative elements; **Table S3.** Comparison of prophages/prophage-like elements and integrative elements integration sites of EcO145 to other STEC strains; **Table S4.** Insertion sequences of the STEC genomes; **Table S5.** Prophage/prophage-like element and integrative element encoded T3SS effectors. Click here for file

Additional file 3: Dataset S1 The 341 non-recombinogenic CDS used in phylogenetic analysis. Click here for file

Additional file 4: Dataset S2 EHEC core and accessory genes. Click here for file

Additional file 5: Dataset S3 Conservation of 177 O157 O-islands in other STEC strains. Click here for file

Additional file 6: Dataset S4 LEE genes in 10 EHEC strains. Click here for file

Additional file 7: Dataset S5 Shiga toxin 2a prophage genes. Click here for file
