# Supplementary material for: Comparative genomics of enterohemorrhagic Escherichia coli O145:H28 demonstrates a common evolutionary lineage with Escherichia coli O157:H7
Source: BMC Genomics. 2014 Jan 10;15:17. doi: 10.1186/1471-2164-15-17 (PMC3893438; doi:10.1186/1471-2164-15-17)
Supplement: Additional file 1: Figure S1 — Genome features of Escherichia coli O145:H28 strain RM13516; Figure S2. Detection of DNA methylation; Figure S3. Gene organization of the three large O-islands (OI) conserved between EcO145 and EcO157; Figure S4. Gene organization and content of the tellurite resistance islands; Figure S5. Gene organization and phylogenetic analysis of LEE island; Figure S6. Gene organization of the two large O-islands encoding T3SS-related proteins; Figure S7. Gene organization and phylogenetic analysis of Stx2a prophage; Figure S8. The chromosomal distribution of STEC major mobile elements; Figure S9. MAFFT and Mauve alignment of EHEC virulence plasmids; and Figure S10. MAFFT and Mauve alignment of EcO145 secondary plasmids. [file 1471-2164-15-17-S1.pdf]

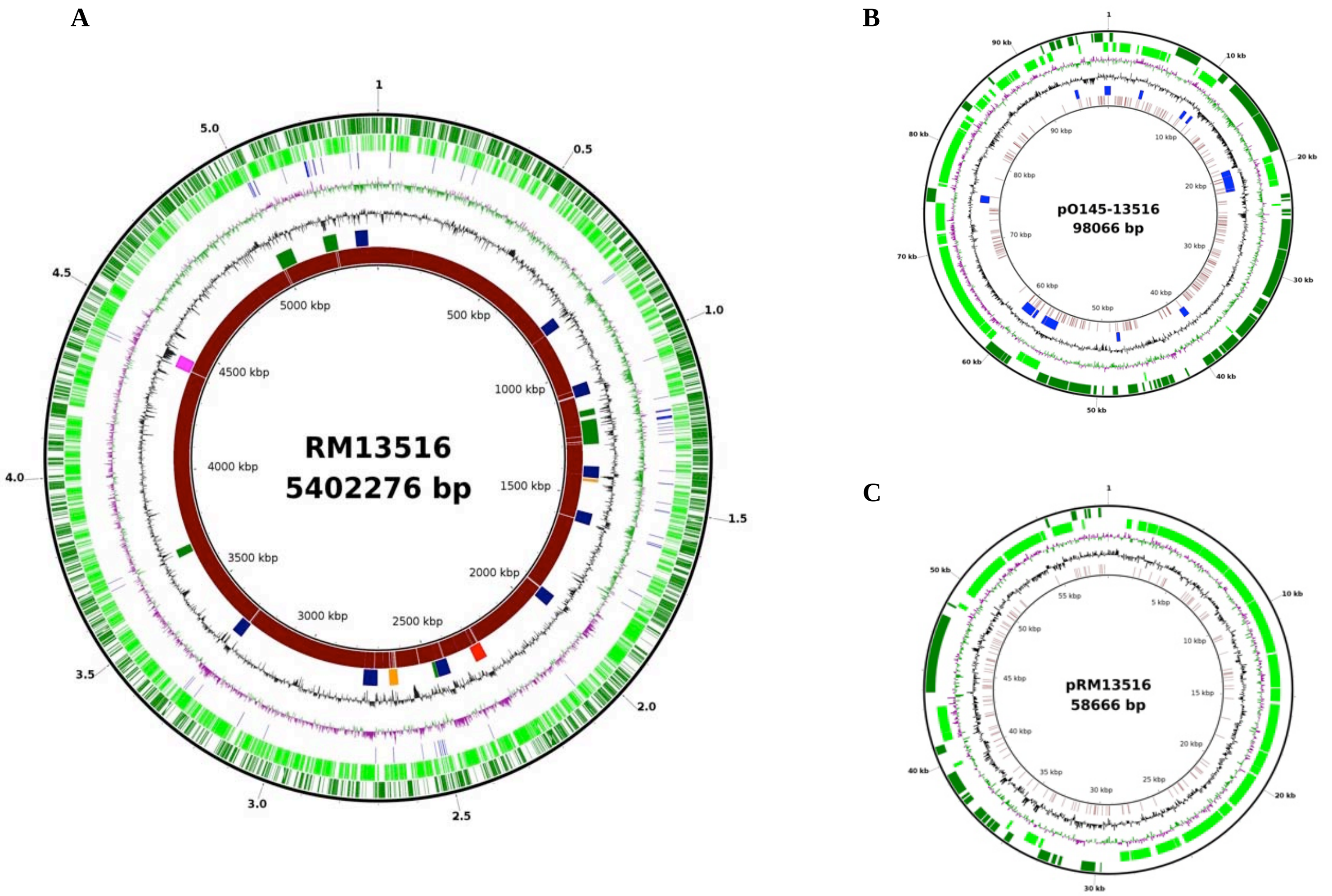


**Figure S1.** Genome features of *Escherichia coli* O145:H28 strain RM13516. Circular maps of the chromosome (**A**), and the two plasmids, pO145-13516 (**B**) and pRM13516 (**C**). **A.** For chromosome, from outer circle to inner circle, each represents positive strand CDS (1), negative strand CDS (2), insertion sequences (3), GC Skew (4), GC Content (5), Prophage/Integrated elements (Prophage – Navy, Prophage-like – Orange, Shiga toxin phage – Red, Integrated element – Green, LEE – pink) (6), and GATC methylation sites (7). **B.** pO145-13516, from outer circle to inner circle, each represents positive strand CDS (1), negative strand CDS (2), GC Skew (3), GC content (4), insertion sequences (5), and GATC methylation sites (6). **C.** pRM13516, from outer circle to inner circle, each represents positive strand CDS (1), negative strand CDS (2), GC Skew (3), GC content (4), and GATC methylation sites (5). The circular maps were generated using BLAST Ring Image Generator (BRIG) software.

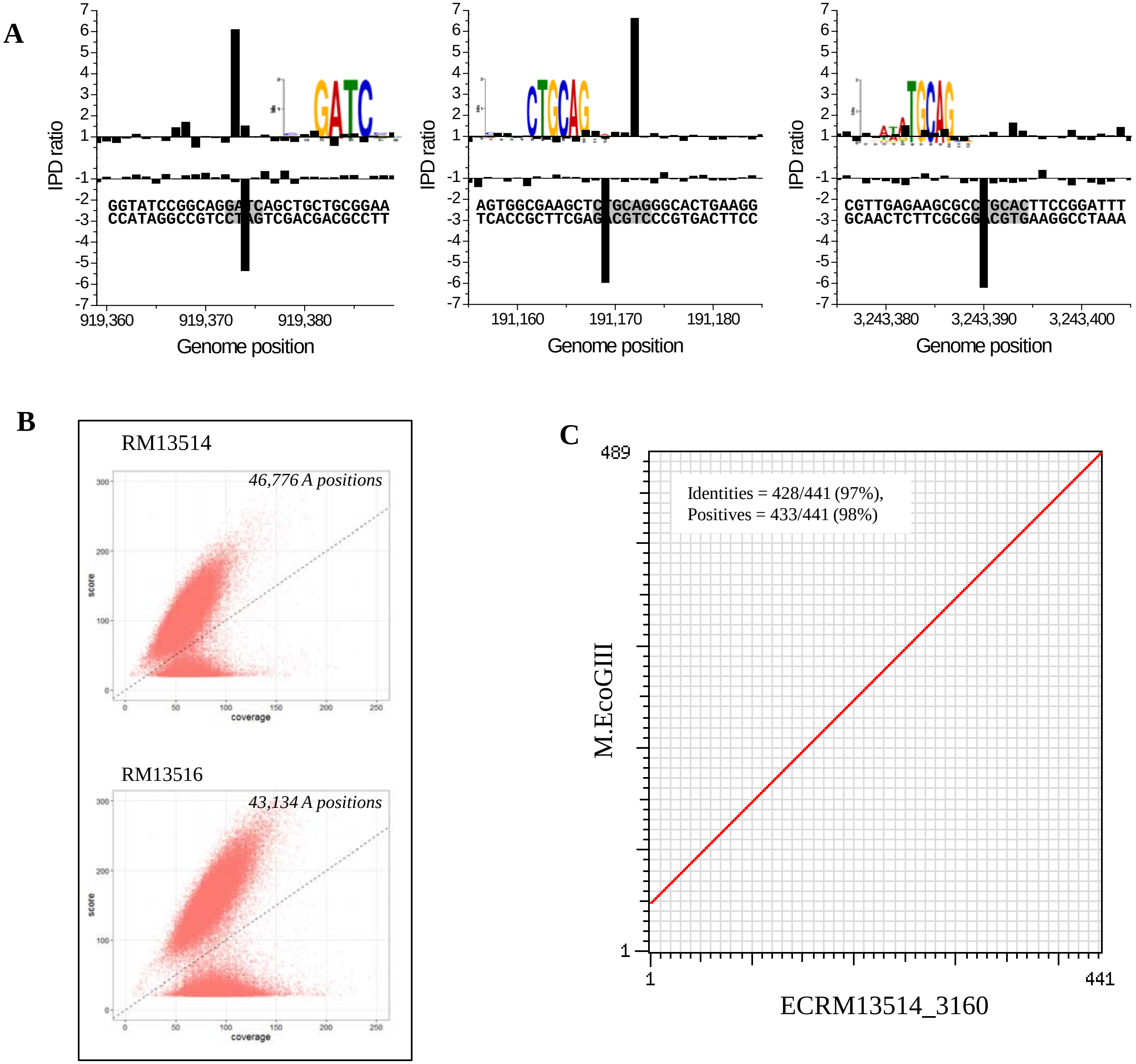


**Figure S2.** Detection of DNA methylation. **A**. motif identification of 5’-Gm6ATC-3’/3’-CTm6AG-5’, motif identification of 5’-CTGCm6AG-3’/3’-Gm6ACGTC-5’, and motif identification of asymmetric, methylation of 5’-CTGCm6AG-3’ based on kinetic signal. **B**. scatter plots of kinetics score versus sequence coverage for RM13514 identifying 46,766 genomic adenines as methylated and for RM13516 identifying 43,134 genomic adenines as methylated. **C**. Alignment of RM13514 *Pst*I methylase with the functionally characterized methylase M.EcoGIII in the STEC O104:H4 strain C227-11. The two proteins have 97% identity across the entire protein encoded by the ECRM13514_3160.

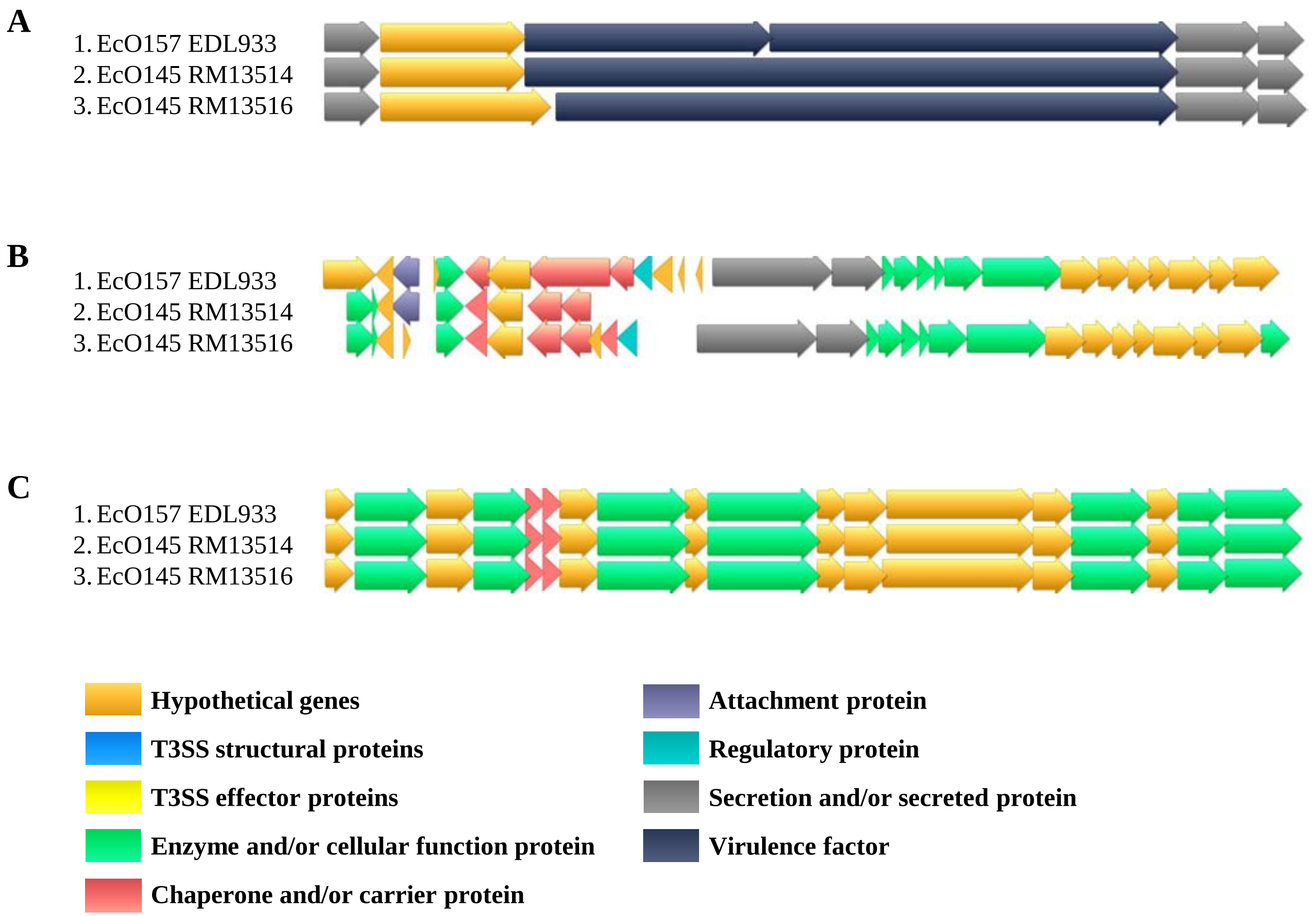


**Figure S3.** Gene organization of the three large O-islands (OI) conserved between EcO145 and EcO157. **A.** OI-28; **B.** OI-47, and **C.** OI-138.

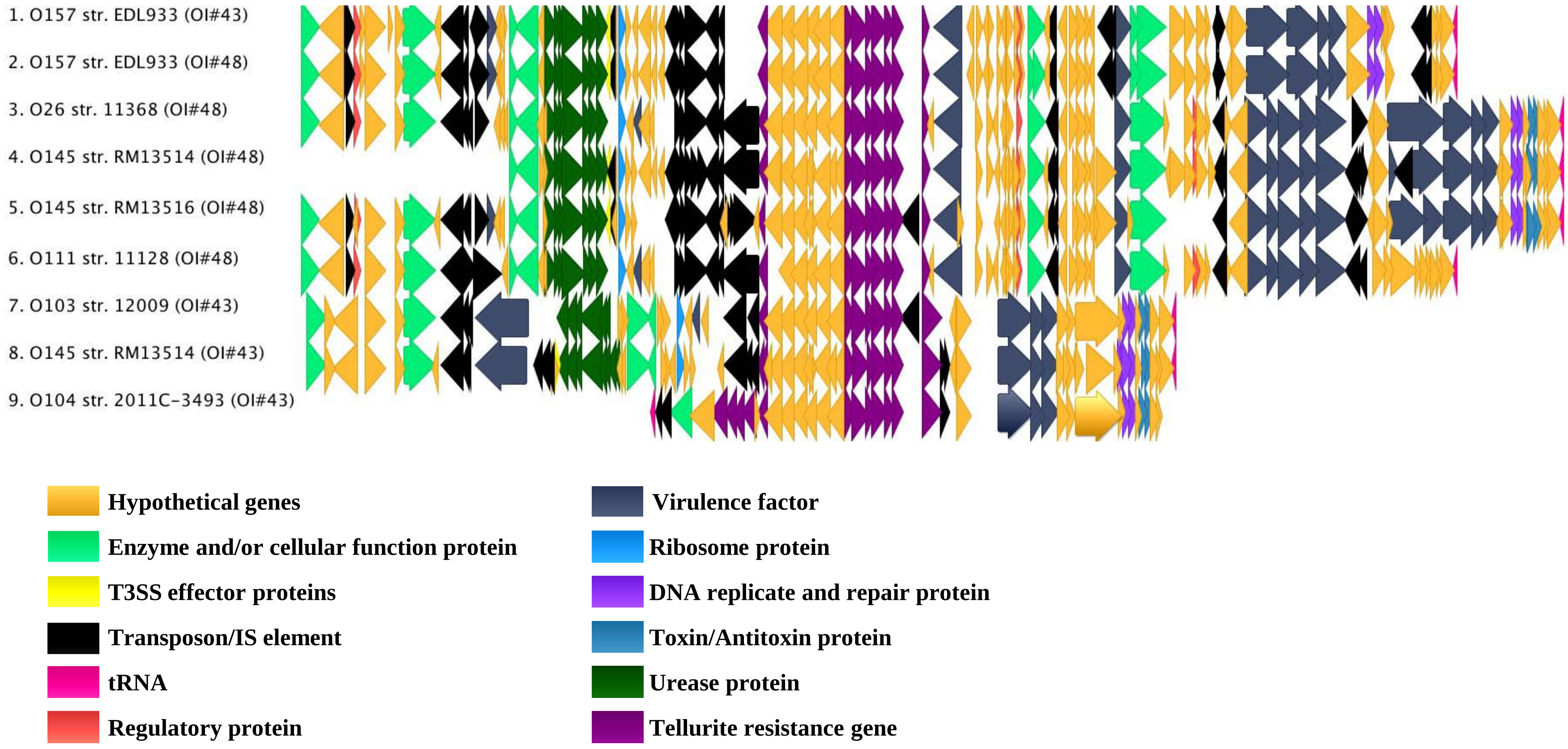


**Figure S4.** Gene organization and content of the tellurite resistance (OI#43 or OI#48) islands for RM13514, RM13516, O103, O26, O111, and O104 compared to EDL933. The tRNA integration site for each region is displayed, and each gene is labeled based on function classification as defined in figure legend. Presence or absence of all O-islands in O145 strains are listed in Dataset S3.

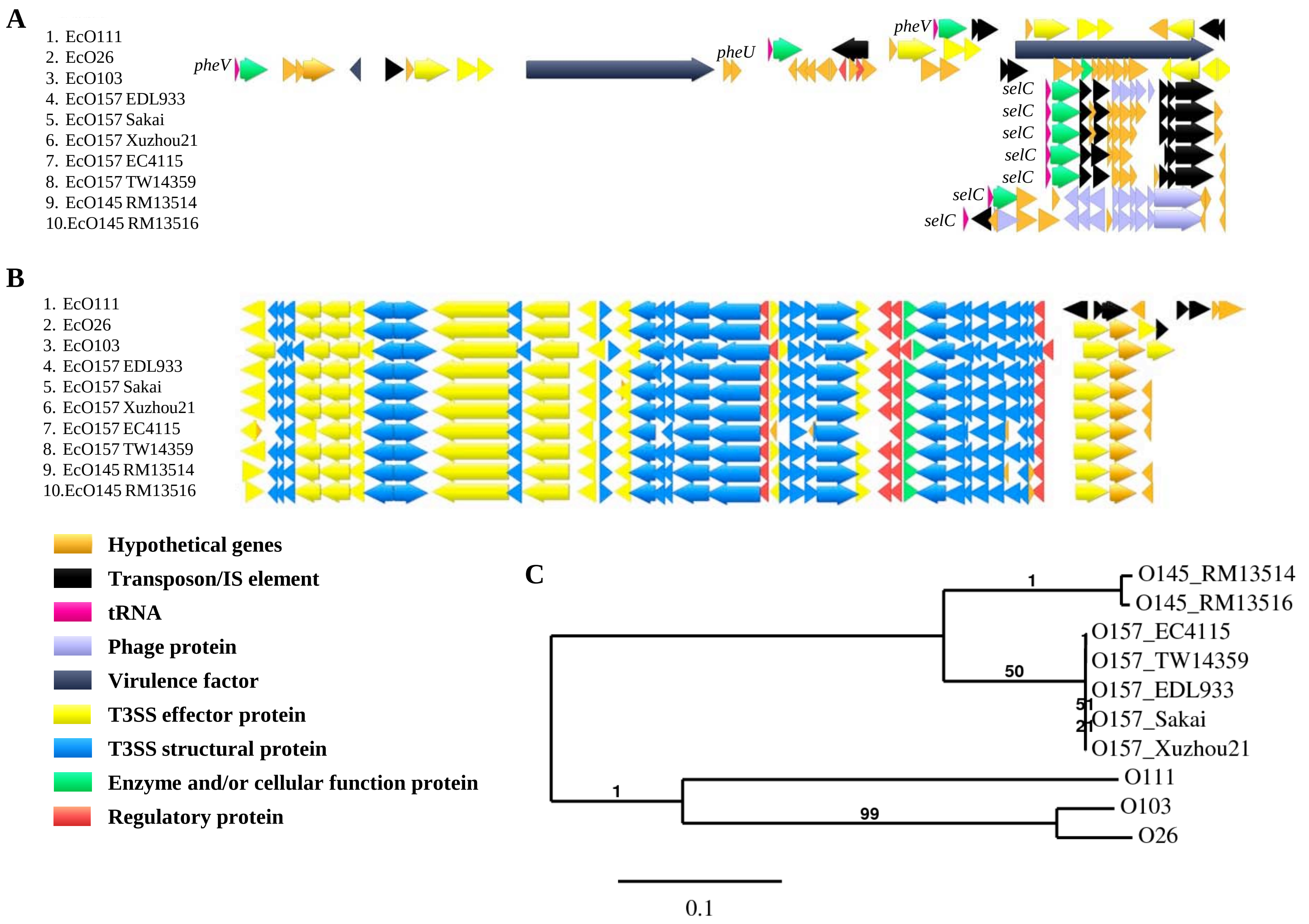


**Figure S5.** Gene organization and phylogenetic analysis of LEE island. **A.** Gene organization of the accessory LEE regions for each of the EHEC strains. The tRNA intergration site for each region is displayed, and each gene is labeled based on function classification as defined in figure legend. Individual genes for each of the strains are listed in Dataset S4. **B.** Gene organization of the core region of the LEE region for each of the EHEC strains. Each gene is labeled based on function classification as defined in figure legend. Individual genes for each of the strains are listed in Supplemental Dataset S4. **C.** Phylogenetic analysis of the LEE region. The nucleotide sequence for each of the LEE regions were aligned using MAFFT, and a maximum likelihood-based tree was generated using RAxML program with the GTR+GAMMA+Invariable sites model and assessed by bootstrapping with 100,000 pseudoreplicates and 100 alternative runs. Scale bar: number of substitutions per base. Branch support values 1 representing 100.

**A**

1. EcO26 str. 11368
2. EcO103 str. 12009
3. EcO111 str. 11128
4. EcO145 str. RM13514
5. EcO145 str. RM13516
6. EcO157 str. EDL933

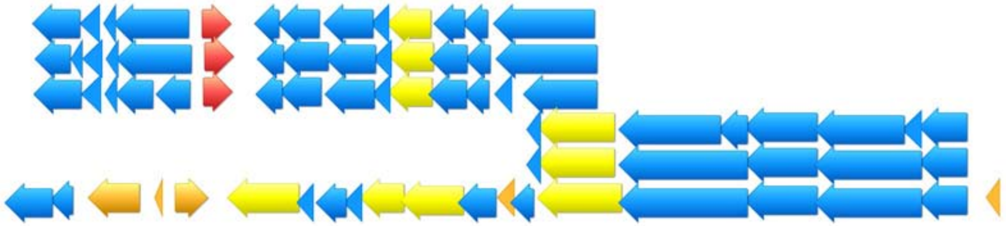

**Hypothetical genes** | **T3SS structural proteins**
**T3SS effector proteins** | **Regulatory protein**

**B**

1. EcO157 str. EDL933
2. EcO103 str. 12009
3. EcO145 str. RM13516
4. EcO145 str. RM13514
5. EcO111 str. 11128

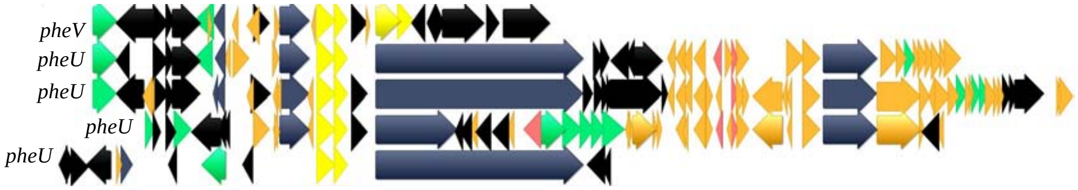


**Hypothetical genes** | **T3SS effector or chaperone protein**
**Transposon/IS element** | **Enzyme or cellular function protein**
**Virulence factor** | **Regulatory protein**

**Figure S6.** Gene organization of the two large O-islands encoding T3SS-related proteins. **A**. Gene organization of OI-115 regions. EcO145 strains contain only ~half the island, while the other non-O157 strains only contain the other half compared with OI-115 of EDL933. **B**. SpLE3-like O-island 122. The tRNA integration site for each region is displayed, and each gene is labeled based on function classification as defined in figure legend. Presence or absence of all O-islands in O145 strains are listed in Dataset S3.

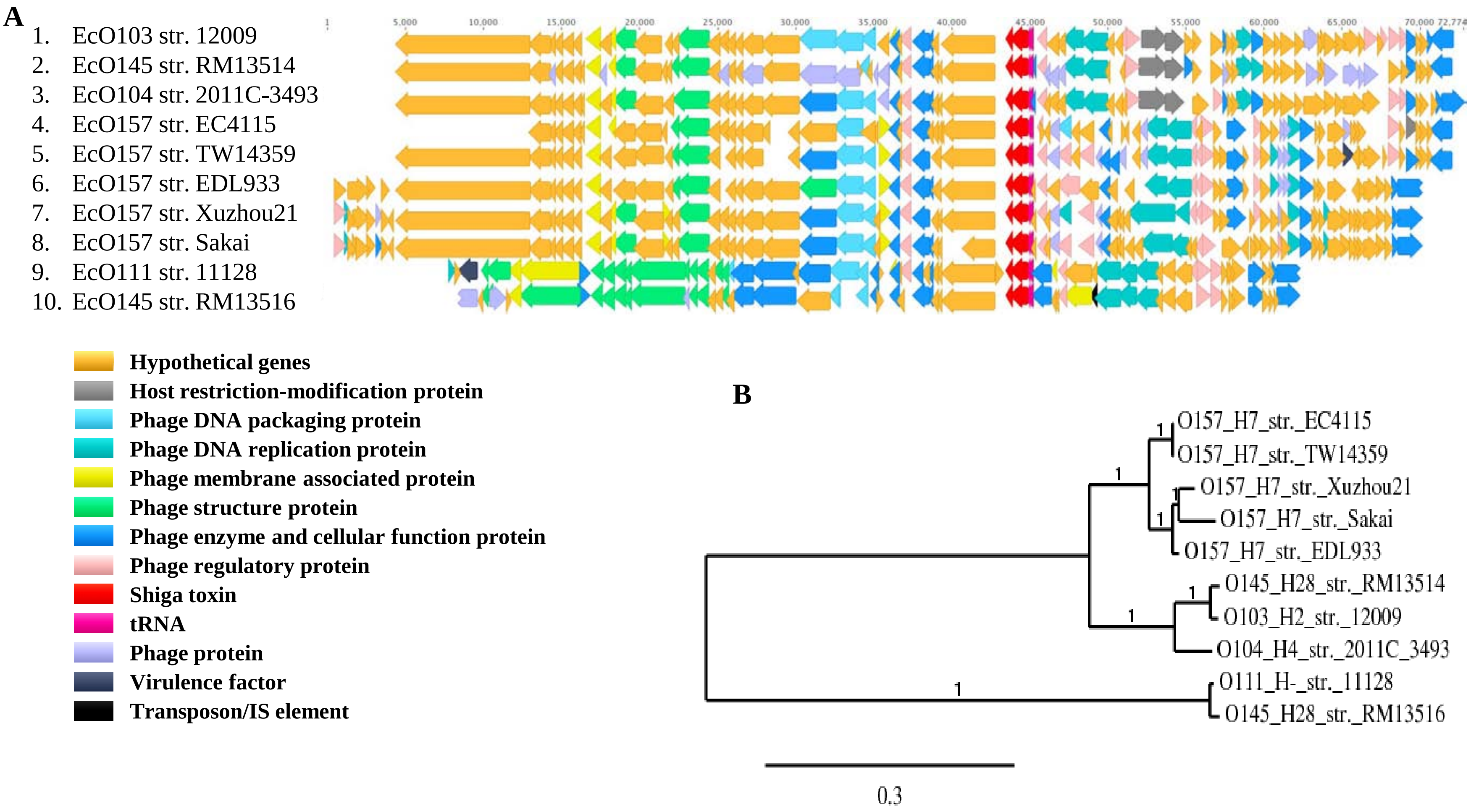


**Figure S7.** Gene organization and phylogenetic analysis of Stx2a prophage. **A.** Gene organization of the Stx2a prophage regions for each of the STEC strains, except for *E. coli* O26:H11 str. 11368 that only has the Stx1 prophage. Each gene is labeled based on function classification as defined in figure legend. Individual genes for each of the strains are listed in Supplemental Dataset S5. **B.** Phylogenetic analysis of the Stx2a prophage region for each of the STEC strains. Stx2a prophage region sequences were aligned using MAFFT, and a maximum likelihood-based tree was generated using the RAxML program with GTR+GAMMA+Invariable sites model and assessed by bootstrapping with 100,000 pseudoreplicates and 100 alternative runs. Scale bar: number of substitutions per base. Branch support values 1 representing 100.

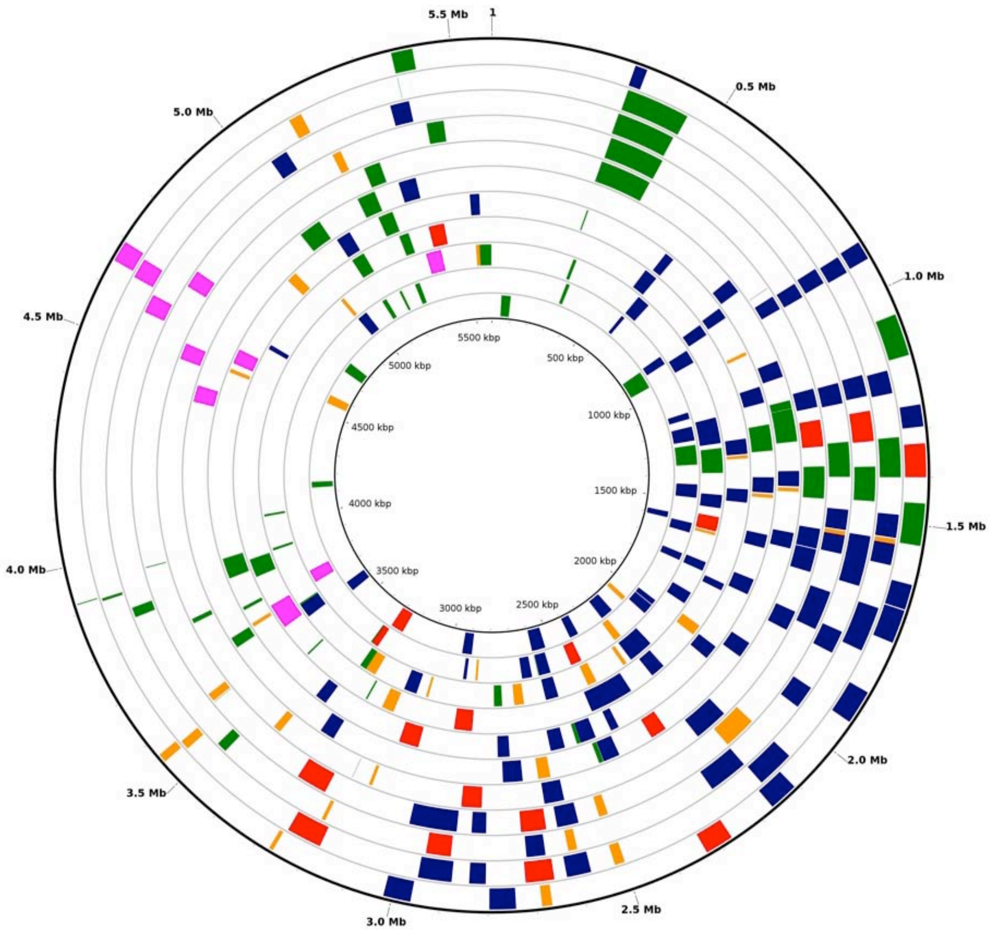


**Figure S8.** The chromosomal distribution of STEC major mobile elements. Prophage (Navy), Shiga toxin genes containing prophage (Red), Prophage-like element (Orange), Integrated element (Green) and LEE (Pink). Outer circle inwards: EcO157 str. EDL933 (1), EcO157 str. TW14359 (2), EcO157 str. Sakai (3), EcO157 str. EC4115 (4), EcO157 str. Xuzhou21 (5), EcO145 str. RM13516 (6), EcO145 str. RM13514 (7), EcO103 str. 12009 (8), EcO26 str. 11368 (9), EcO111 str. 11128 (10), and Ec O104 str. 2011C-3493 (11).

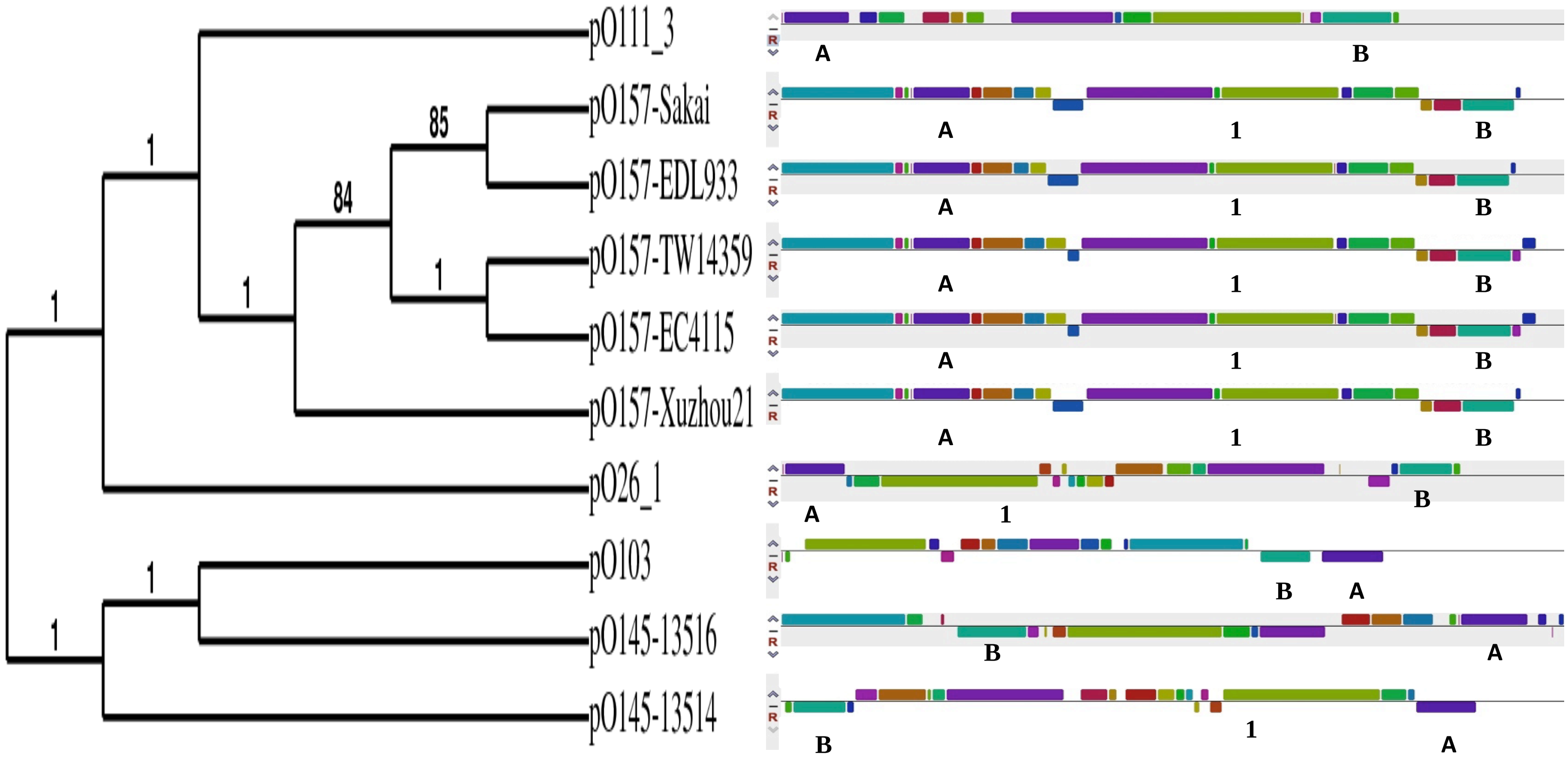


**Figure S9.** MAFFT and Mauve alignment of EHEC virulence plasmids. The complete nucleotide sequence s of EHEC virulence plasmids were aligned by the MAFFT program and displayed by maximum likelihood-based tree constructed with the RAxML program using the GTR+GAMMA+Invariable sites model (left panel). Branch support values 1 representing 100. The Mauve alignments exhibit re-arrangements between the virulence plasmids. Region A represents the gene cluster related to production of enterohemolysin and region B represents the gene cluster related to lipid A modification . Region 1 represents the gene encoding large toxin ToxB. Similar color indicates the conserved region among various plasmids.

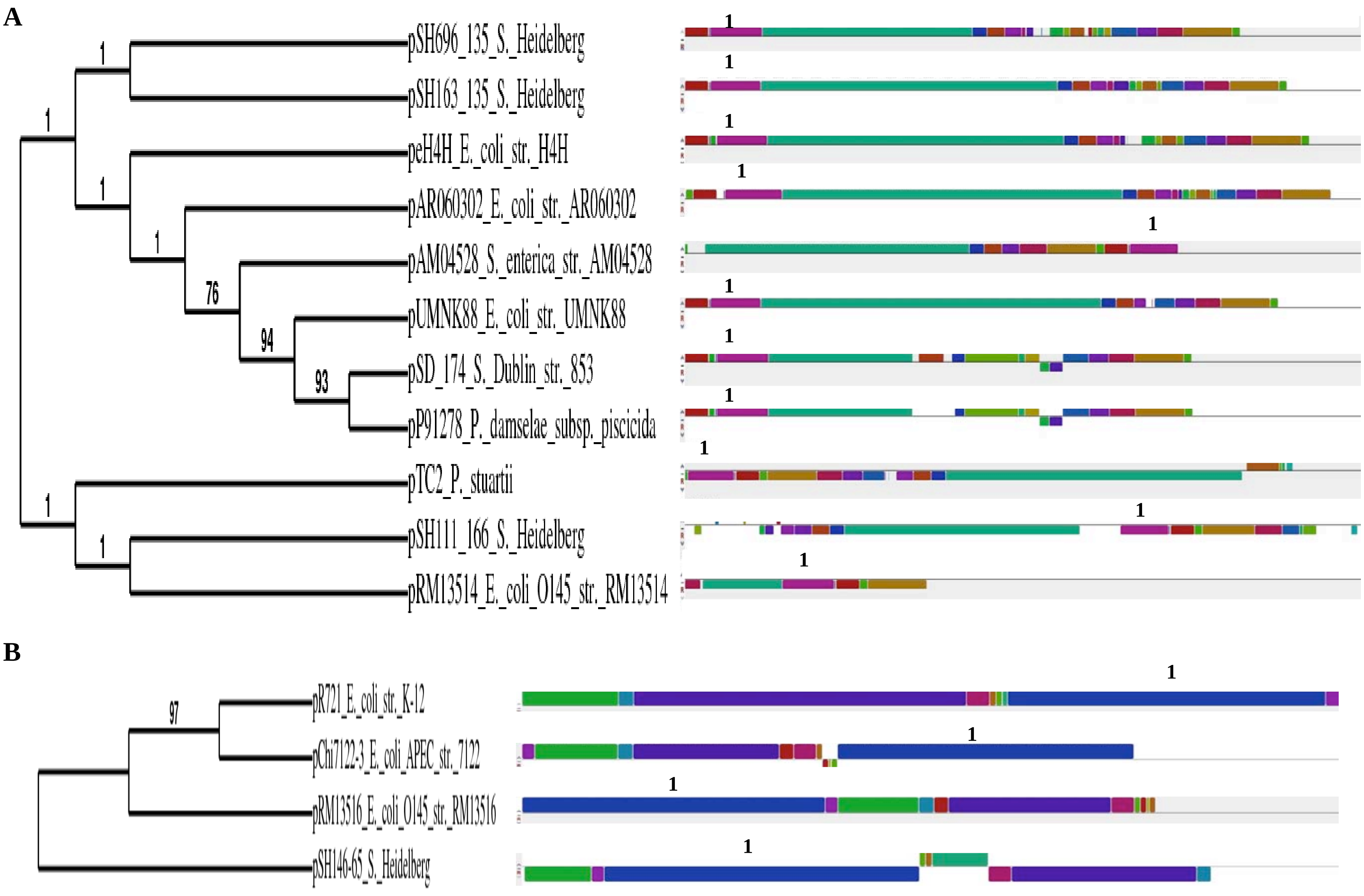


**Figure S10. MAFFT and Mauve alignment of EcO145 secondary plasmids. A.** MAFFT alignment of the EcO145 plasmid pRM13514 with the ten closest plasmid sequences and displayed by maximum likelihood-based tree constructed with RAxML program based on a MAFFT alignment of the complete nucleotide sequence using the GTR+GAMMA+Invariable sites model (left panel), and Mauve alignments of the 10 plasmids (right panel). Branch support values 1 representing 100. The region 1 represents genes conferring cell the multidrug resistances. **B**. MAFFT alignment of the EcO145 plasmid pRM13516 with the closest plasmid sequences and displayed maximum likelihood-based tree constructed with RAxML program using the GTR+GAMMA+Invariable sites model (left panel), and Mauve alignments exhibit re-arrangements between the plasmids (right panel). The region labeled 1 represents genes encodes for the type IVb pilus and VirB-related type IV secretion system. Similar color indicates the conserved region among various plasmids.
