# Supplementary material for: Comparative genomics of enterohemorrhagic Escherichia coli O145:H28 demonstrates a common evolutionary lineage with Escherichia coli O157:H7
Source: BMC Genomics. 2014 Jan 10;15:17. doi: 10.1186/1471-2164-15-17 (PMC3893438; doi:10.1186/1471-2164-15-17)
Supplement: Additional file 2: Table S1 — EcO145 strain-specific genes and their functional categories; Table S2.E. coli O145:H28 prophage/prophage-like elements and integrative elements; Table S3. Comparison of prophages/prophage-like elements and integrative elements integration sites of EcO145 to other STEC strains; Table S4. Insertion sequences of the STEC genomes; Table S5. Prophage/prophage-like element and integrative element encoded T3SS effectors. [file 1471-2164-15-17-S2.docx]

Table S1. EcO145 strain-specific genes and their functional categories.

| **Locus_Tag** | **Length (a.a.)** | **Function** | **Functional Category** |
| --- | --- | --- | --- |
| **RM13514** | | | |
| ECRM13514_0282 | 330 | Aldo-keto reductase | Metabolism |
| ECRM13514_0283 | 284 | Oxidoreductase | Metabolism |
| ECRM13514_0284 | 1418 | Putative adhesin | Attachment |
| ECRM13514_0285 | 214 | Transcriptional regulator YkgA | Regulation |
| ECRM13514_0286 | 290 | 2,5-diketo-D-gluconic acid reductase | Metabolism |
| ECRM13514_0287 | 198 | Membrane protein YkgB | Membrane protein |
| ECRM13514_0289 | 442 | Putative Dihydrolipoamide dehydrogenase | Metabolism |
| ECRM13514_0290 | 285 | Putative HTH-type transcriptional regulator YkgD | Regulation |
| ECRM13514_0292 | 240 | Predicted L-lactate dehydrogenase, subunit YkgE | Metabolism |
| ECRM13514_0293 | 476 | Predicted L-lactate dehydrogenase, subunit YkgF | Metabolism |
| ECRM13514_0294 | 232 | Predicted L-lactate dehydrogenase, subunit YkgG | Metabolism |
| ECRM13514_0582 | 318 | Protease VII (Omptin) precursor | Enzymes |
| ECRM13514_0996 | 1382 | Cell division protein FtsK | Cell division |
| ECRM13514_1207 | 271 | Putative regulator | Regulation |
| ECRM13514_1212 | 279 | Outer membrane usher protein FimD | Outer membrane protein |
| ECRM13514_1469 | 40 | Leucine-rich repeat protein |  |
| ECRM13514_1745 | 220 | Beta-phosphoglucomutase | Metabolism |
| ECRM13514_1885 | 89 | Protein transcriptional regulator HipB | Regulation |
| ECRM13514_1887 | 188 | Type 1 fimbriae major subunit FimA | Outer membrane protein |
| ECRM13514_2053 | 265 | Putative protein kinase | Regulation |
| ECRM13514_2429 | 228 | Putative metal-binding protein domain SecA | Secretion |
| ECRM13514_2430 | 405 | Type I restriction-modification system, subunit S | DNA modification |
| ECRM13514_2431 | 384 | Type I restriction-modification system, DNA-methyltransferase subunit M | DNA modification |
| ECRM13514_2656 | 46 | Exodeoxyribonuclease I | DNA |
| ECRM13514_2657 | 44 | Error-prone repair protein UmuD | DNA |
| ECRM13514_2666 | 85 | Putative secreted protein | Secretion |
| ECRM13514_2714 | 202 | Essential recombination function protein Erf | DNA |
| ECRM13514_2764 | 465 | Colanic acid biosynthesis protein WcaM | Membrane protein |
| ECRM13514_3117 | 93 | Putative outer membrane protein | Outer membrane protein |
| ECRM13514_3149 | 51 | Putative DNA methylase | DNA |
| ECRM13514_3159 | 490 | Modification methylase PstI | DNA modification |
| ECRM13514_3160 | 318 | Type II restriction enzyme BsuBI | DNA |
| ECRM13514_3182 | 209 | Adenine DNA methyltransferase | DNA modification |
| ECRM13514_3309 | 182 | Hydrogenase-4 component H | Metabolism |
| ECRM13514_3868 | 54 | Restriction endonuclease, GTPase subunit | DNA |
| ECRM13514_3974 | 593 | Retron-type reverse transcriptase | DNA synthase |
| ECRM13514_3999 | 155 | TerW | Tellurite resistance |
| ECRM13514_4020 | 1330 | Serine protease autotransporter enterotoxin EspC | Protease |
| ECRM13514_4027 | 569 | tRNA-dihydrouridine synthase | RNA |
| ECRM13514_4645 | 95 | Predicted transcriptional regulator Pch-homolog | Regulation |
| ECRM13514_4685 | 93 | Type III secretion protein SsaH | Secretion |
| ECRM13514_4994 | 313 | DNA-cytosine methyltransferase | DNA modification |
| ECRM13514_5190 | 163 | Putative methyltransferase | DNA modification |
| ECRM13514_5488 | 84 | Programmed cell death antitoxin ChpS | Toxin/Anti-toxin |
| ECRM13514_5489 | 117 | Programmed cell death toxin ChpB | Toxin/Anti-toxin |
| ECRM13514_5535 | 60 | Putative ATP-dependent protease | Protease |
| ECRM13514_5540 | 499 | Putative membrane protein | Membrane protein |
| ECRM13514_5544 | 953 | Putative ATP-dependent helicase | DNA |
| ECRM13514_5545 | 1212 | Type II restriction enzyme, methylase subunit | DNA modification |
| ECRM13514_5548 | 258 | Type II restriction enzyme, methylase subunit | DNA modification |
| ECRM13514_5549 | 2114 | Helicase | DNA |
| ECRM13514_5550 | 705 | Putative DNA helicase | DNA |
| ECRM13514_5580 | 1747 | Adherence and invasion outer membrane protein | Membrane protein |
| **RM13516** | | | |
| ECRM13516_0650 | 65 | Phosphopantetheinyltransferase | Biosynthesis |
| ECRM13516_0931 | 1304 | Cell division protein FtsK | Cell division |
| ECRM13516_1143 | 160 | Putative chaperone protein | Resistance |
| ECRM13516_1144 | 187 | Fimbrial subunit CupA1 | Adhesin |
| ECRM13516_1145 | 1271 | ShlA/HecA/FhaA exofamily protein | Toxin |
| ECRM13516_1146 | 540 | ShlA/HecA/FhaA exofamily protein | Toxin |
| ECRM13516_1147 | 123 | Holo-[acyl-carrier protein] synthase | Biosynthesis |
| ECRM13516_1148 | 254 | 3-oxoacyl-[acyl-carrier protein] reductase | Metabolism |
| ECRM13516_1149 | 183 | Putative fatty acyl chain dehydrase | Metabolism |
| ECRM13516_1150 | 91 | Acyl carrier protein | Metabolism |
| ECRM13516_1151 | 387 | Putative aminomethyltransferase | Biosynthesis |
| ECRM13516_1152 | 853 | Putative 3-oxoacyl-[acyl-carrier-protein] synthase synthase | Biosynthesis |
| ECRM13516_1153 | 408 | Malonyl CoA-acyl carrier protein transacylase | Biosynthesis |
| ECRM13516_1155 | 236 | ABC transporter, ATP-binding protein | Transport |
| ECRM13516_1160 | 315 | Malonyl CoA-acyl carrier protein transacylase | Biosynthesis |
| ECRM13516_1165 | 83 | Putative regulatory protein | Regulation |
| ECRM13516_1526 | 508 | Retron-type reverse transcriptase | RNA |
| ECRM13516_1709 | 219 | Beta-phosphoglucomutase | Metabolism |
| ECRM13516_2019 | 42 | Beta-lactam resistance protein | Resistance |
| ECRM13516_2336 | 276 | Membrane protease | Enzyme |
| ECRM13516_2537 | 1158 | Adherence and invasion outermembrane protein | Outer membrane protein |
| ECRM13516_2557 | 405 | Putative ribokinase | Metabolism |
| ECRM13516_2560 | 345 | Puative phophotriesterase | Enzyme |
| ECRM13516_2561 | 38 | Gamma-glutamyltranspeptidase | Enzyme |
| ECRM13516_2562 | 56 | Gamma-glutamyltranspeptidase | Enzyme |
| ECRM13516_3166 | 147 | Hydrogenase-4 component H | Metabolism |
| ECRM13516_3357 | 1502 | Pertactin precursor |  |
| ECRM13516_3732 | 278 | Type II/IV secretion system ATPase TadZ/CpaE | Secretion/transporter |
| ECRM13516_3734 | 423 | Type II/IV secretion system secretin RcpA/CpaC | Secretion/transporter |
| ECRM13516_4041 | 181 | Similar to C-terminal Zn-finger domain of DNA topoisomerase I | DNA |
| ECRM13516_4451 | 44 | Replication protein O | DNA |
| ECRM13516_4845 | 180 | Heat shock protein C | Resistance |
| ECRM13516_5069 | 121 | Queuosine biosynthesis QueD | Biosynthesis |
| ECRM13516_5072 | 1505 | Lhr-like helicase | DNA |
| ECRM13516_5076 | 817 | ATP-dependent DNA helicase RecQ | DNA |
| ECRM13516_5077 | 969 | RNA polymerase associated protein RapA | RNA |
| ECRM13516_5079 | 417 | Archaeosine tRNA-ribosyltransferase type 5 | RNA |
| ECRM13516_5220 | 178 | Putative virulence-related membrane protein | Membrane |
| ECRM13516_5368 | 71 | Cold shock protein CspH | Resistance |
| ECRM13516_5369 | 72 | Cold shock protein CspB | Resistance |
| ECRM13516_5374 | 71 | Cold shock-like protein CspI | Resistance |

**Table S2.** *E. coli* O145:H28 prophage/prophage-like elements and integrative elements

| **Name** | **Position (start)** | **Position (end)** | **Length (bp)** | **Integration site** | **Predicted phage (type)** | **T3SS effectors/Shiga toxin** |
| --- | --- | --- | --- | --- | --- | --- |
| **RM13514 - Prophages** |  |  |  |  |  |  |
| Prophage 01 | 596,570 | 626,550 | 29,981 | *envY* | Enterobacteria lambda phage (lambda-like) |  |
| Prophage 02 | 838,469 | 874,226 | 35,758 | *ybhC – ybhB* | Stx2-converting phage 1717 (lambda-like) | *espJ, cif, nleG, nleH* |
| Prophage 03 | 998,887 | 1,009,345 | 10,459 | *cspD – clpS* | Enterobacteria phage P4 (P2-like) |  |
| Prophage 04 | 1,117,160 | 1,168,910 | 51,751 | tRNA (serT) | Enterobacteria phage BP-4795 (lambda-like) | *espV, tccP* |
| Prophage 05 | 1,407,947 | 1,457,248 | 49,302 | *potC - potB* | Stx2-converting phage 1717 (lambda-like) | *espK, espN, espO, espX, nleB* |
| Prophage 06 | 1,462,241 | 1,473,357 | 11,117 | *ycfD – phoQ* | *Shigella flexneri* bacteriophage V (Myoviridae) |  |
| Prophage 07 | 1,591,323 | 1,633,674 | 42,352 | *ompW* | Stx2-converting phage 1717 (lambda-like) | *espO, espM, nleG, nleG* |
| Prophage 08 | 1,738,593 | 1,789,019 | 50,427 | *ydaO – ydfJ* | Enterobacteria lambda phage (lambda-like) |  |
| Prophage 09 | 1,982,031 | 2,027,763 | 45,733 | *ynaF - rspB* | Enterobacteria phage VT1 (unclassified) | *nleC* |
| Prophage 10 | 2,382,743 | 2,409,068 | 26,326 | tRNA (leuZ) | Enterobacteria phage P2 (P2-like) |  |
| Prophage 11 | 2,461,624 | 2,511,124 | 49,501 | tRNA (serU) | Enterobacteria lambda phage (lambda-like) | *nleB, nleH, nleF* |
| Prophage 12 | 2,565,255 | 2,610,389 | 45,135 | *sbcB* | Enterobacteria phage HK97 (lambda-like) |  |
| Prophage 13 | 2,743,631 | 2,780,882 | 37,252 | *yehU – yehV* | Enterobacteria phage BP-4795 (lambda-like) | *espV, nleA, nleH* |
| Prophage 14 | 3,021,368 | 3,083,886 | 62,519 | tRNA (argW) | Stx2-converting phage I (Podoviridae) | *stx2A, stx2B* |
| Prophage 15 | 3,358,887 | 3,396,516 | 37,630 | *ssrA - ypjA* | Enterobacteria phage VT1 (unclassified) | *espS, nleG,* |
| Prophage 16 | 3,690,577 | 3,702,220 | 11,644 | *yggA* | Enterobacteria phage P4 (P2-like) |  |
| Prophage 17 | 4,527,589 | 4,539,582 | 11,994 | *yicC – dinD* | Enterobacteria phage P4 (P2-like) |  |
| Prophage 18 | 4,871,945 | 4,905,354 | 33,410 | *cpxP - fieF* | Yersinia phage L-413C (P2-like) |  |
| Prophage 19 | 5,073,571 | 5,119,969 | 46,399 | *dusA* | Stx2-converting phage 1717 (lambda-like) | *espS, nleG* |
| Prophage 20 | 5,520,929 | 5,550,561 | 29,633 | *yjjG - prfC* | Enterobacteria phage cdtI (lambda-like) |  |
| **RM13514 – Integrative elements** | | | | | | |
| IE_01 | 311,682 | 315,759 | 4,078 | tRNA (argU) | Hypothetical proteins |  |
| IE_02 | 1,239,492 | 1,322,952 | 83,461 | tRNA (serX) | Tellurite resistance | *espC* |
| IE_03 | 2,512,334 | 2,524,767 | 12,434 | tRNA (asnT) | Type VI secretion system effectors |  |
| IE_04 | 3,750,708 | 3,761,008 | 10,301 | tRNA (pheV) | Iron transport system |  |
| IE_05 | 3,861,137 | 3,926,474 | 65,338 | tRNA (metX) | Tellurite resistance | *espC* |
| IE_06 | 4,558,936 | 4,605,536 | 46,601 | tRNA (selC) | LEE | *espA, espB, espD, espF, espG, espH, espZ, map, tir, eae* |
| IE_07 | 5,222,118 | 5,269,766 | 47,649 | tRNA (pheU) | O-island #122 | *espL, nleB, nleE* |
| **RM13516 - Prophages** | | | | | | |
| Prophage 01 | 788,599 | 831,762 | 43,164 | *ybhC - ybhB* | *Salmonella typhimurium* phage Gifsy-1 (unclassified) | *espJ, nleG, nleH, cif* |
| Prophage 02 | 1,063,508 | 1,112,165 | 48,658 | tRNA(serT) | Enterobacteria phage BP-4795 (lambda-like) | *espV, tccP* |
| Prophage 03 | 1,389,289 | 1,433,714 | 44,426 | *potC - potB* | Enterobacteria phage BP-4795 (lambda-like) | *espK, espN, espO, espX, nleB* |
| Prophage 04 | 1,438,787 | 1,449,888 | 11,102 | *ycfD – phoQ* | *Shigella flexneri* bacteriophage V (Myoviridae) |  |
| Prophage 05 | 1,570,124 | 1,615,548 | 45,425 | *ompW* | Stx2-converting phage 1717 (lambda-like) | *espM, espO, nleG, nleG* |
| Prophage 06 | 1,915,329 | 1,962,979 | 47,651 | *ydfJ - rspB* | Enterobacteria lambda phage (lambda-like) | *nleC* |
| Prophage 07 | 2,263,973 | 2,311,142 | 47,170 | *yecD - yecE* | Enterobacteria phage BP-4795 (lambda-like) | *stx2A, stx2B, nleC* |
| Prophage 08 | 2,417,842 | 2,465,975 | 48,134 | tRNA (serU) | Enterobacteria lambda phage (lambda-like) | *nleB, nleF, nleH* |
| Prophage 09 | 2,627,127 | 2,662,865 | 35,739 | *yegQ - yegR* | Enterobacteria phage P2 (P2-like) |  |
| Prophage 10 | 2,708,790 | 2,765,911 | 57,122 | *yehU - yehV* | Enterobacteria phage BP-4795 (lambda-like) | *espS, espV, nleA, nleG* |
| Prophage 11 | 3,279,903 | 3,323,347 | 43,445 | *ssrA - ypjA* | Stx2-converting phage 1717 (lambda-like) | *espS, nleG* |
| Prophage 12 | 5,317,104 | 5,367,177 | 50,074 | *yjjG - prfC* | Enterobacteria phage cdtI (lambda-like) |  |
| **RM13516 – Integrated Elements** | | | | | | |
| IE_01 | 1,181,905 | 1,205,665 | 23,761 | *ycdU* | Part of O-island #47 |  |
| IE_02 | 1,207,048 | 1,302,914 | 95,867 | tRNA (serX) | Tellurite resistance | *espC* |
| IE_03 | 2,467,129 | 2,480,809 | 13,681 | tRNA (asnT) | Type VI secretion system effectors |  |
| IE_04 | 3,665,936 | 3,697,973 | 32,038 | tRNA (pheV) | Iron transport system, O-island #95, Type II/IV secretion system |  |
| IE_05 | 4,410,578 | 4,458,362 | 47,785 | tRNA (selC) | LEE | *espA, espB, espD, espF, espG, espH, espZ, map, tir, eae* |
| IE_06 | 4,994,108 | 5,060,401 | 66,194 | tRNA (leuX) | Toxin/anti-toxin system, iron transport system |  |
| IE_07 | 5,188,896 | 5,242,157 | 53,262 | tRNA (pheU) | O-island #122 | *espL, nleB, nleE* |

**Table S3.** Comparison of prophages/prophage-like elements and integrative elements integration sites of EcO145 to other STEC strains

| **O145 strains^*^** | **O157 strains** | | | | | **non-O157 strains** | | | |
| --- | --- | --- | --- | --- | --- | --- | --- | --- | --- |
|  | EDL933 | Sakai | TW14539 | EC4115 | Xuzhou21 | O26 | O103 | O111 | O104 |
| *envY* |  |  |  |  |  | X | X | X |  |
| *ybhC-ybhB* | X | X | X | X |  | X | X | X | X |
| *cspD-clpS* |  |  |  |  |  |  |  |  |  |
| *serT* | X | X | X |  | X | X |  | X |  |
| *potC-potB* |  | X | X | X | X | X | X | X |  |
| *ycfD-phoQ* | X | X | X |  | X | X | X |  |  |
| *ompW* |  | X | X | X | X | X | X | X | X |
| *ydaO-ydfJ* | X | X |  |  |  | X | X | X |  |
| *ynaF-rspB* |  |  |  |  |  |  |  |  |  |
| *leuZ* | X |  |  |  |  | X |  |  |  |
| *serU* | X | X |  | X | X |  | X | X |  |
| *sbcB* |  |  | X | X |  |  |  | X |  |
| *yehU-yehV* | X | X | X |  | X |  |  |  |  |
| *argW* | X | X | X | X | X | X | X |  | X |
| *ssrA-ypjA* |  | X |  |  |  | X | X | X | X |
| *yggA* |  |  |  |  |  |  |  |  |  |
| *yicC-dinD* |  |  |  |  |  |  | X |  |  |
| *cpxP-fieF* |  |  |  |  |  |  |  |  |  |
| *dusA* |  |  | X | X |  |  |  |  |  |
| *yjjG-prfC* |  |  |  |  |  |  | X |  |  |
| *argU* |  |  | X | X |  | X | X | X |  |
| *asnT* |  |  |  |  |  | X | X |  | X |
| *pheV* | X | X | X | X | X | X | X | X | X |
| *metX* |  |  |  |  |  |  | X |  |  |
| *selC* | X | X | X | X | X | X |  |  | X |
| *pheU* |  |  |  |  |  | X | X | X | X |
| *yecD-yecE* |  |  |  |  |  |  | X | X |  |
| *yegQ-yegR* |  |  | X |  |  |  |  |  |  |
| *phoH* |  |  |  |  |  |  |  |  |  |
| *serX* | X | X | X | X | X | X |  | X |  |
| *leuX* | X | X | X | X | X | X | X | X | X |
| *ycdU* | X |  |  |  |  |  |  |  |  |

^*^ Prophage/prophage-like elements and integrative elements chromosomal integration sites of both RM13514 and RM13516

**Table S4.** Insertion sequences of the STEC genomes^a^

| IS Element | **O145 strains** | | O157 strains | | | | | non-O157 strains | | | |
| --- | --- | --- | --- | --- | --- | --- | --- | --- | --- | --- | --- |
|  | **RM13514** | **RM13516** | EDL933 | Sakai | EC4115 | Xuzhou21 | TW14359 | O103 | O26 | O111 | O104 |
| IS1 | **2** | **4** | 2 | 1 | 3 | 1 | 2 | 1 | 4 | 6 (4) | 11 (1) |
| IS1 (NuXi) | **0** | **1** | 3 (2) | 6 (2) | 3 (1) | 4 (2) | 2 | 4 | 4 | 5 | 1 |
| IS2 | **2** | **0** | 1 | 4 | 1 | 1 | 0 | 4 | 2 | 5 | 0 |
| IS3 | **0** | **1** | (2) | 4 (2) | (2) | (2) | (2) | 5 (2) | 4 (1) | 3 (1) | 1 |
| IS4 family IS element | **0** | **0** | 0 | 0 | 0 | 0 | 0 | 0 | 1 | 1 | 0 |
| IS5 | **0** | **0** | 0 | 0 | 0 | 0 | 0 | 0 | 0 | 0 | 7 (3) |
| IS10 | **0** | **0** | 0 | 0 | 0 | 0 | 0 | 0 | 0 | (1) | 0 |
| IS15 | **(3)** | **0** | 0 | 0 | 0 | 0 | 0 | 0 | 0 | 0 | 0 |
| IS21 | **0** | **0** | 0 | (1) | 0 | 0 | 0 | 2 | 0 | 0 | 0 |
| IS26 | **0** | **0** | 0 | 0 | 0 | 0 | 0 | 0 | 0 | (4) | 3 |
| IS30 | **0** | **2** | 6 | 5 | 4 | 3 | 4 | 0 | 3 | 3 | 2 |
| IS91 | **(3)** | **(4)** | (1) | (1) | (1) | (1) | (1) | 0 | (4) | 5 (3) | 0 |
| IS100 | **0** | **0** | 0 | 0 | 0 | 0 | 0 | 0 | 0 | 0 | 3 |
| IS102 | **0** | **0** | 0 | 2 | 0 | 0 | 0 | 4 | 2 | 2 | 0 |
| IS110/IS492 | **0** | **0** | 0 | 0 | 0 | 0 | 0 | 0 | 1 | 0 | 0 |
| IS200 | **0** | **0** | 0 | 0 | 0 | 0 | 0 | 0 | (1) | (1) | 1 |
| IS421 | **0** | **0** | 0 | 0 | 0 | 0 | 0 | 0 | 0 | 0 | 4 |
| IS600 | **11 (2)** | **1** | 4 (1) | 3 | 3 (1) | 3 (1) | 3(1) | 0 | 10 (2) | 1 | 5 (1) |
| IS602 | **2** | **3 (2)** | 3 | 2 | 2 | 4 (1) | 1 | 1 | 0 | 4 | 1 |
| IS604 | **2** | **2 (1)** | (1) | 4 | (1) | 3 | 5 (1) | 2 | 2 | 3 | 0 |
| IS608 | **1** | **1** | 4 | 1 | 3 | 1 | 1 | 2 | 1 | 3 | 0 |
| IS609 | **2** | **2** | 2 | 4 | 2 | 2 | 2 | 2 | 3 | 5 | 4 |
| IS617 | **0** | **0** | 0 | 1 | 1 | 2 | 1 | 0 | 0 | (1) | 0 |
| IS621 | **0** | **0** | 0 | 0 | 0 | 0 | 0 | 12 | 14 | 2 | 9 |
| IS629 | **37 (6)** | **19 (2)** | 23 (2) | 23 (4) | 22 (3) | 20 (2) | 23 (3) | 34 (7) | 13 (5) | 49 (12) | 11 (2) |
| IS630 | **2** | **0** | 2 | 2 | 0 | 2 | 2 | 2 | 2 | 3 | (1) |
| IS677 | **1** | **1** | 6 | 7 | 5 | 5 | 0 | 7 | 6 | 6 | 5 |
| IS679 | **0** | **0** | 0 | 0 | 0 | 0 | 0 | 0 | 0 | 0 | (1) |
| IS682 | **2 (1)** | **2** | 4 | 5 | 3 | 3 | 3 | 9 | 7 (5) | 3 | 8 (4) |
| IS683 | **0** | **0** | 1 | 1 | 1 | 1 | 0 | 2 | (3) | 1 | 0 |
| IS688 | **1** | **0** | 3 | 2 | 1 | 1 | 1 | 0 | 0 | 0 | 2 |
| IS911 | **4 (1)** | **3 (1)** | (1) | (1) | (1) | (1) | (1) | 5 (2) | 2 (1) | 2 (1) | 2 |
| IS1151 | **0** | **0** | 0 | 1 | 0 | 0 | 0 | 0 | 1 | 1 | 0 |
| IS1397 | **0** | **0** | 0 | 0 | 0 | 0 | 0 | 0 | 0 | 1 | 0 |
| IS1414 | **1** | **2** | 4 | 0 | 4 | 4 | 4 | (1) | 3 (1) | 3 (2) | 2 |
| ISCro1 | **0** | **0** | 0 | 0 | 0 | 0 | 0 | 3 | 11 | 1 | 0 |
| ISCro3 | **1** | **0** | 0 | 0 | 0 | 0 | 0 | 0 | 0 | 0 | 0 |
| ISEc8 | **7 (2)** | **11 (2)** | 12 | 11 | 12 (1) | 11 | 12 (1) | 9 (1) | 9 (4) | 7 (3) | 8 (1) |
| ISEc16 | **0** | **0** | 0 | 0 | 0 | 0 | 0 | 0 | 1 | 0 | 0 |
| ISEc17 | **(3)** | **0** | 0 | 0 | 0 | 0 | 0 | 0 | 0 | 0 | 0 |
| ISEc18 | **1** | **0** | 2 | 0 | 1 | 1 | 1 | 0 | 0 | 0 | 1 |
| ISEc20 | **6** | **6** | 0 | 0 | 0 | 0 | 0 | 1 | 0 | 1 | 0 |
| ISEc21 | **0** | **0** | 0 | 0 | 0 | 0 | 0 | 0 | 0 | 0 | 1 |
| ISEc22 | **2 (1)** | **2 (1)** | 0 | 0 | 0 | 0 | 0 | 1 | 7 | 0 | 0 |
| ISEc31 | **0** | **(1)** | 0 | 0 | 0 | 0 | 0 | 0 | 0 | 0 | 0 |
| ISEc33 | **0** | **2** | 0 | 0 | 0 | 0 | 0 | 0 | 0 | 0 | 2 |
| ISEc38 | **3 (2)** | **1** | 0 | 0 | 0 | 0 | 0 | 0 | 0 | 0 | 3 (1) |
| ISEc43 | **0** | **0** | 0 | 0 | 0 | 0 | 0 | 0 | 0 | 0 | 0 |
| ISEc47 | **0** | **2** | 4 | 0 | 0 | 0 | 0 | 0 | 0 | 0 | 0 |
| ISEc48 | **1** | **2 (1)** | 1 | 0 | 0 | 0 | 0 | 0 | 0 | 0 | (2) |
| ISEc49 | **6** | **2** | 2 | 0 | 1 | 1 | 2 | 0 | 0 | 0 | 0 |
| ISEcp1 | **0** | **0** | 0 | 0 | 0 | 0 | 0 | 0 | 0 | 0 | (1) |
| ISErsp1 | **0** | **0** | 1 | 0 | 1 | 1 | 1 | 0 | 0 | 0 | 1 |
| ISSba14 | **1** | **1** | 0 | 0 | 0 | 0 | 0 | 0 | 0 | 0 | 0 |
| ISSfl2 | **1** | **0** | 0 | 0 | 0 | 0 | 0 | 0 | 0 | 0 | 0 |
| ISSfl3 | **9 (4)** | **2** | 1 | 1 | 0 | 0 | 1 | 0 | 1 | 0 | 0 |
| ISSfl8 | **0** | **0** | 0 | 0 | 0 | 0 | 0 | 0 | 0 | 0 | (1) |
| ISSfl10 | **2** | **3** | 0 | 0 | 0 | 0 | (1) | 0 | 0 | 0 | 0 |
| ISSfl11 | **(4)** | **0** | 0 | 0 | 0 | 0 | 0 | 0 | 0 | 0 | 0 |
| ISShes11 | **0** | **0** | 0 | 0 | 0 | 0 | 0 | 0 | 0 | 0 | 1 |
| ISStma11 | **0** | **0** | 0 | 0 | 0 | 0 | 0 | 0 | 0 | 0 | 1 |
| ISVsa3 | **(1)** | **0** | 0 | 0 | 0 | 0 | 0 | 0 | 0 | 0 | 0 |
| ISSwi1 | **0** | **0** | 0 | 0 | 0 | 0 | 0 | 0 | 0 | 0 | (2) |
| Total | **124 (33)** | **89 (16)** | 95 (10) | 92 (11) | 84 (12) | 78 (10) | 75 (10) | 113 (13) | 122 (27) | 133 (32) | 108 (21) |

^a^Insertion sequences present in plasmids are in parentheses

**Table S5.** Prophage/prophage-like element and integrative element encoded T3SS effectors^a^

| Effectors | **O145 strains** | | O157 strains | | | | | non-O157 strains | | | |
| --- | --- | --- | --- | --- | --- | --- | --- | --- | --- | --- | --- |
|  | **RM13514** | **RM13516** | EDL933 | Sakai | EC4115 | Xuzhou21 | TW14359 | O103 | O26 | O111 | O104 |
| *espA* | **1** | **1** | 1 | 1 | 1 | 1 | 1 | 1 | 1 | 1 | 0 |
| *espB* | **1** | **1** | 1 | 1 | 1 | 1 | 1 | 1 | 1 | 1 | 0 |
| *espC* | **2** | **1** | 0 | 0 | 0 | 0 | 0 | 1 | 0 | 0 | 3 |
| *espD* | **1** | **1** | 1 | 1 | 1 | 1 | 1 | 1 | 1 | 1 | 0 |
| *espF* | **1** | **1** | 1 | 1 | 1 | 1 | 1 | 1 | 1 | 1 | 0 |
| *espG* | **1** | **1** | 1 | 1 | 1 | 1 | 1 | 1 | 1 | 1 | 0 |
| *espH* | **1** | **1** | 1 | 1 | 1 | 1 | 1 | 1 | 1 | 1 | 0 |
| *espJ* | **1** | **1** | 1 | 1 | 1 | 1 | 1 | 0 | 1 | 1 | 0 |
| *espK* | **1** | **1** | 1 | 1 | 1 | 1 | 1 | 3 | 2 | 1 | 0 |
| *espL* | **1** | **1** | 1 | 1 | 1 | 1 | 1 | 2 | 1 | 2 | 0 |
| *espM* | **1** | **1** | 2 | 2 | 2 | 2 | 2 | 2 | 2 | 2 | 0 |
| *espN* | **1** | **1** | 1 | 1 | 1 | 1 | 1 | 1 | 1 | 1 | 0 |
| *espO* | **2** | **2** | 2 | 2 | 2 | 2 | 2 | 2 | 2 | 2 | 0 |
| *espP* | **1** | **0** | 1 | 1 | 1 | 1 | 1 | 0 | 0 | 1 | 1 |
| *espR* | **1** | **1** | 1 | 1 | 1 | 1 | 1 | 1 | 1 | 1 | 1 |
| *espS* | **2** | **2** | 0 | 0 | 1 | 0 | 1 | 2 | 2 | 1 | 0 |
| *espT* | **0** | **0** | 0 | 0 | 0 | 0 | 0 | 0 | 0 | 0 | 0 |
| *espV* | **2** | **2** | 1 (1) | 1 (1) | 1 (1) | 1 (1) | 1 (1) | 1 (1) | 1 (1) | 1 (1) | 0 |
| *espW* | **0** | **0** | 1 | 1 | 1 | 1 | 1 | 1 | 1 | 1 | 0 |
| *espX* | **1** | **1** | 1 | 1 | 1 | 1 | 1 | 1 | 1 | 1 | 0 |
| *espZ* | **1** | **1** | 1 | 1 | 1 | 1 | 1 | 1 | 1 | 1 | 0 |
| *map* | **1** | **1** | 1 | 1 | 1 | 1 | 1 | 1 | 1 | 1 | 0 |
| *nleA/espI* | **1** | **1** | 1 | 1 | 1 | 1 | 1 | 1 | 1 (1) | 1 | 0 |
| *nleB* | **3** | **3** | 3 (1) | 3 (1) | 3 (1) | 3 (2) | 3 (1) | 4 | 1 | 2 | 0 |
| *nleC* | **2 (1)** | **3 (1)** | 1 | 1 | 1 | 1 | 1 | 2 (2) | 1 | 2 | 0 |
| *nleD* | **0** | **0** | 1 | 1 | 1 | 1 | 1 | 0 | 0 | 0 | 0 |
| *nleE* | **1** | **1** | 1 | 1 | 1 | 1 | 1 | 2 | 1 | 2 | 0 |
| *nleF* | **1** | **1** | 1 | 1 | 1 | 1 | 1 | 1 | 1 | 1 (1) | 0 |
| *nleG* | **6 (1)** | **7 (2)** | 15 | 14 (6) | 16 (2) | 12 (1) | 15 (1) | 8 (2) | 14 | 11 (3) | 0 |
| *nleH* | **3** | **2** | 2 | 2 | 2 | 2 | 2 | 2 (1) | 2 | 2 | 0 |
| *tccP* | **1** | **1** | 2 | 2 (1) | 2 (1) | 2 | 2 | 1 | 1 | 1 | 0 |
| *tir* | **1** | **1** | 1 | 1 | 1 | 1 | 1 | 1 | 1 | 1 | 0 |
| *cif* | **1** | **1** | 0 | 0 | 0 | 0 | 0 | 1 (1) | 1 (1) | 1 (1) | 0 |
| Total | **44 (2)** | **43 (3)** | 49 (2) | 48 (9) | 51 (5) | 46 (4) | 50 (3) | 48 (7) | 47 (3) | 47 (6) | 5 (0) |

^a^Pseudogenes are shown in parentheses
